# Phylogenetics within *Camassia* (Asparagaceae): examining difficult taxonomy and unusual variation using genomic restriction-site-associated DNA sequencing data

**DOI:** 10.7717/peerj.20438

**Published:** 2026-01-14

**Authors:** Jenny K. Archibald, Susan R. Kephart, Patrick J. Monnahan, Kathryn E. Theiss, Theresa M. Culley

**Affiliations:** 1 Undergraduate Biology Program, University of Kansas, Lawrence, KS, United States of America; 2 Department of Biology, Willamette University, Salem, OR, United States of America; 3 Adaptive Biotechnologies, Seattle, WA, United States of America; 4 Department of Biology, California State University, Dominguez Hills, Carson, CA, United States of America; 5 Department of Biological Sciences, University of Cincinnati, Cincinnati, OH, United States of America

**Keywords:** Asparagaceae, *Camassia*, Divergence, *Hastingsia*, Phylogeny, Progenitor-derivative, RADseq, Subspecies, Taxonomy

## Abstract

**Premise:**

Diversification of *Camassia* (Asparagaceae) in North America has shaped variety in morphological, ecological, and reproductive traits, and resulted in a classification with ambiguity in taxon boundaries, including numerous putative subspecies. Phylogenetic analyses of restriction-site-associated DNA sequences (RADseq) allowed new insights into its evolution and taxonomy, enhancing understanding of basal relationships, geographic patterns, taxonomic boundaries, and potential new species.

**Methods:**

A total of 157 individuals in 71 populations across all 15 putative taxa of *Camassia* and 42 outgroup individuals in 21 populations from *Hastingsia* and *Chlorogalum* were sampled and genomic libraries were generated using the modified single-digest RADseq method known as multiplexed shotgun genotyping. Assembly in ipyrad included targeted comparisons across a range of parameters that influence homology assessment and amount of missing data, with analysis of the set of resulting datasets in RAxML and SVDquartets followed by comparison and summary across the sets of trees.

**Results and Conclusions:**

Increasing the number of sampled loci improved phylogenetic signal despite concurrent increases in missing data. Each taxon was generally cohesive on the phylogenies, but some species and subspecies were not monophyletic. Results suggest that there was an early separation of *C. howellii* and *C. leichtlinii* from the rest of the genus during diversification. Different analysis parameters supported either a clade of both species as sister to the remainder of the genus, or *C. howellii* alone as sister. Relationships among all relatively deep clades within the genus were well supported. Within species, *C. leichtlinii* had particularly robust support for relationships compared to others in *Camassia*, and geographic patterns corresponding to the diversification of some subclades were resolved. The eight subspecies of *C. quamash* largely formed two main clades. Although most subspecies showed sufficient phylogenetic, morphological, or ecological distinctiveness to maintain recognition, we recommend synonymizing *C. quamash* ssp. *intermedia* into *C. quamash* ssp.* maxima*. Some outlier individuals of *C. quamash* were resolved with other species. For example, the status of *C. quamash* ssp.* utahensis* was called into question by a well-supported division placing two populations with *C. cusickii*. In the disjunct species complex distributed further east in North America (*C. scilloides*+), results confirmed at least one progenitor-derivative species pair (*C. angusta* arising from *C. scilloides*) and some evidence for a potential new taxon closely related to *C. angusta*. Overall, these taxa have diversified in traits that can result in genetic isolation, such as differences in flowering seasons and ecological preferences, but there also are indications of some continued gene flow among both subspecies and species.

## Introduction

**Figure 1:**
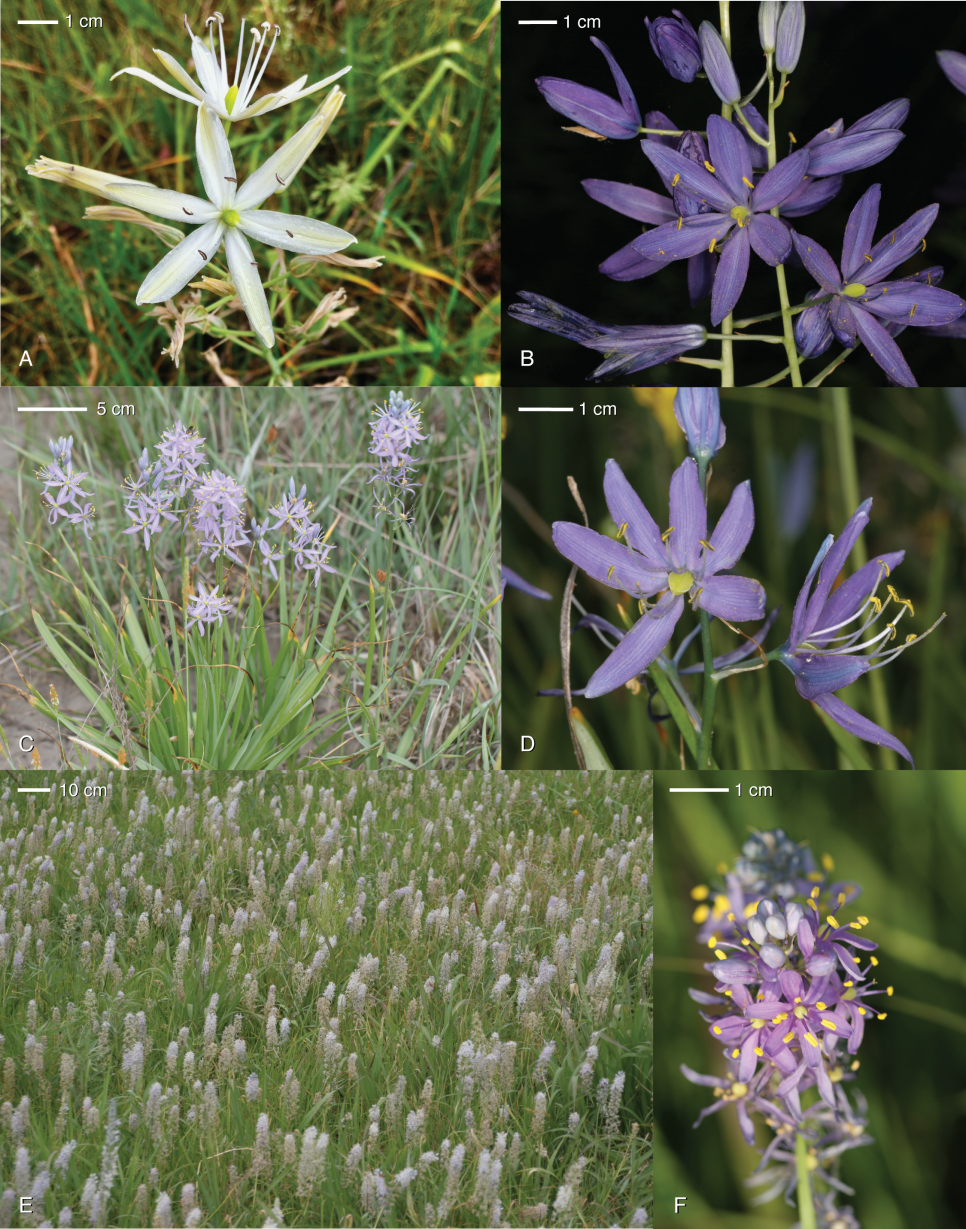
Examples of morphology across *Camassia*. (A) *C. leichtliniii* ssp. *leichtlinii* population PS, (B) *C. leichtlinii* ssp. *suksdorfii* FC, (C) *C. quamash* ssp. *walpolei* KR, (D) *C. quamash* ssp. *maxima* MDF, (E) a prairie with individuals of *C. scilloides* BAR, and (F) *C. angusta* OSA. See Appendix I for an explanation of population codes. Photo credit: JK Archibald.

The extraordinary diversity of taxa and characters across the tree of life also reflects a diversity of mechanisms that allowed those lineages to diverge (De Queiroz, 2007). A thorough understanding of this diversity requires integration of phylogenetic, morphological, and ecological information, which is known as an integrative or iterative taxonomy approach to species delimitation (Dayrat, 2005; Padial & De La Riva, 2010; Yeates et al., 2011). As a case study for this type of approach, we are examining the plant genus *Camassia* Lindl. (Fig. 1; Asparagaceae; *e.g.*, Kephart et al., 2025). Despite its tractable size of six named species, this genus encompasses diversity of various types, connected to a surprisingly complex taxonomy (Gould, 1942; Ranker & Hogan, 2002). Distributed within North America, some endemic species range across less than 100 km^2^, while others spread beyond 1,000 km^2^. The generic distribution encompasses a disjunction; four species are found in the Western USA and adjacent regions in Canada with a center of diversity in the Northwest US (*C. cusickii* S. Watson, *C. howellii* S. Watson, *C. leichtlinii* (Baker) S. Watson, and *C. quamash* (Pursh) Greene), while two species occur farther east: in the Midwest and southern US (*C. angusta* (Engelm. & A. Gray) Blank. and *C. scilloides* (Raf.) Cory; Ranker & Hogan, 2002). The two disjunct groups are separated by over 1,300 km at their closest point. One species (*C. quamash*) has eight subspecies (Gould, 1942), raising questions as to why such putative infraspecific diversity occurs without full separation into species and making application of taxon names difficult in the field. Morphological distinctions among subspecies and even among some species are not always clear (Kephart, 2015). Taxa in this genus also vary in ecological preferences (such as edaphic differences and prairie *vs.* wooded habitats; Merritt, 2021) and reproductive traits (such as in diurnal anthesis timing and flowering season; Archibald pers. obs., 2013; Ranker & Schnabel, 1986; Watson, 1889). There is hybridization between some infrageneric taxa and reproductive barriers between others (Uyeda & Kephart, 2006). Varying components (geography, morphology, ecology, and reproductive traits) allow examination of differing potential paths for diversification, likely having roles in the current levels of genetic isolation or the initial divergence of taxa (Coyne & Orr, 2004). A clearer understanding of taxonomy is also valuable for conservation efforts, such as in confirming that the locally appropriate taxon is used in restoration efforts (Kephart, 2015) and identifying distinct evolutionary lineages that may benefit from independent management plans. Many of these taxa sustain pollinator, herbivore, and human communities, historically and today (Carney et al., 2021; Parachnowitsch & Elle, 2005; Sultany, Kephart & Eilers, 2007).

This paper provides a robust estimate of phylogeny as a key tool for further investigations of evolution within *Camassia* and for informing work to organize its diversity into a usable and meaningful classification. We also employed the phylogenetic results in combination with other knowledge on the biology of these organisms to examine specific unresolved taxonomic and phylogenetic questions in the genus, described below. Prior phylogenetic work on this group includes broader analyses in Agavoideae (Asparagaceae, APG, 2009; APG, 2016; Chase, Reveal & Fay, 2009). Analyses of one nuclear and two chloroplast loci by Archibald et al. (2015) and analyses of four chloroplast regions by Halpin & Fishbein (2013) strongly demonstrated the reciprocal monophyly and sister relationship of *Camassia* and *Hastingsia* S. Watson, with *Chlorogalum* Kunth s.s. (Taylor & Keil, 2018) sister to the *Camassia*–*Hastingsia* clade. Fishbein et al. (2010) provided the first phylogenetic trees across *Camassia*, sequencing two chloroplast regions and sampling at least one member of each species and subspecies, and the phylogenetic framework produced by Archibald et al. (2015) had particular focus on *Camassia* and its sister genus *Hastingsia*. The two studies supported different roots for *Camassia*, which could alter infrageneric appraisals of character evolution and biogeography. Although these prior studies provided a significant step forward in understanding evolutionary patterns within *Camassia*, many questions remain, from the basal rooting of the genus to relationships among and within species.

One species of interest is *C. leichtlinii*. Most treatments recognized it as a separate species (Gould, 1942; McNeal, 2012; Ranker & Hogan, 2002), and allozyme analyses suggested that *C. leichtlinii* and *C. quamash* largely remain genetically distinct. However, many sites show close sympatry between the two species, and some hybrid individuals have been identified (Uyeda & Kephart, 2006). Covering an extensive latitudinal range from southern Canada through California in the US, *C. leichtlinii* occurs both near and far from other species of *Camassia*. A more expansive study across populations of this widespread species would help clarify relationships with other western *Camassia*.

Moreover, relationships remain unclear among the eight subspecies of the other widespread western species, *C. quamash*. Both Fishbein et al. (2010) and Archibald et al. (2015) inferred two main clades of *C. quamash* individuals: a *C. q. azurea*+ clade comprised of *C. quamash* subspecies *azurea* (A. Heller) Gould, *intermedia* Gould, *linearis* Gould, *maxima* Gould, and *walpolei* (Piper) Gould, and a *C. q. breviflora*+ clade comprised of subspecies *breviflora* Gould, *quamash*, and *utahensis* Gould (Archibald et al., 2015). Yet, instead of being placed with other *C. quamash* in prior phylogenies, several populations of *C. q. utahensis* were placed with another species instead (*C. cusickii*). Overall, many relationships within these two clades were unresolved.

Difficult questions also remain in the more eastern *C. scilloides* complex, which may include two derivative species along with its namesake. There has been long-standing disagreement on whether *C. angusta* should be recognized as a separate species from *C. scilloides* (Gould, 1942; Steyermark, 1961). Ranker & Schnabel (1986) integrated studies of allozyme diversity, morphology, and phenology of this pair and concluded they were separate species. Those authors recommended a phylogenetic test of whether *C. angusta* is a derivative of *C. scilloides*, but sampling in prior phylogenetic studies was insufficient to address this question. Additionally and more recently, a new potential derivative taxon (referred to as *C.* “glade”) was discovered in Arkansas (Merritt, 2021), and it has not yet been included in a phylogenetic study.

Here we contribute the first in-depth phylogenetic analyses that include multiple samples and multiple populations from each putative infrageneric taxon, employing genome-level molecular markers. As a reduced-representation genomic sequencing approach, restriction-site-associated DNA sequencing (RADseq; Andolfatto et al., 2011; Baird et al., 2008) supplies many variable markers across the genome without requiring full genomic sequences, often allowing insights into even rapidly evolving clades, such as those within the *Camassia* species complexes. This method has previously proven useful for answering a range of biological questions in non-model species (Andrews et al., 2016). Its utility for inferring phylogenetic relationships among closely related taxa has been supported by multiple studies (Anderson et al., 2024; Cariou, Duret & Charlat, 2013; Chase, Stankowski & Streisfeld, 2017; Cruaud et al., 2014; Eaton & Ree, 2013; Frajman et al., 2019; Hipp et al., 2014; Leaché & Oaks, 2017; Marincek et al., 2024; Massatti, Reznicek & Knowles, 2016; Rubin, Ree & Moreau, 2012; Wagner et al., 2013). Although a loss of signal is seen with greater age, a simulation study by Rubin, Ree & Moreau (2012) supported utility of RADseq for phylogeny inference in clades as old as 40–60 myo. The *Camassia* clade is relatively young, estimated at approximately 5.22 million years old (myo) while a *Camassia*–*Chlorogalum* clade was estimated to be 6.85 myo (Smith et al., 2008; *Hastingsia* was not sampled).

In addition to providing a more robust phylogenetic framework across the genus through increased population and locus sampling, we specifically focused on these unresolved questions: (1) What are the basal relationships and root within *Camassia*? (2) In *C. leichtlinii*, do phylogenetic patterns vary with geography and are they affected by sympatry with congeners? (3) How do relationships within and among the many subspecies of *C. quamash* relate to their biology and taxonomy? (4) What is the status of *C. quamash* ssp. *utahensis* and *C. cusickii* in terms of their monophyly and potential sister relationship? and (5) For the more eastern taxa, we asked: (a) Where is the *C. scilloides* complex placed relative to the rest of *Camassia*, (b) is *C. angusta* supported as a derivative of *C. scilloides*, and (c) what is the placement of the potential new taxon *C.* “glade”?

## Materials & Methods

### Taxon sampling and DNA extraction

**Figure 2:**
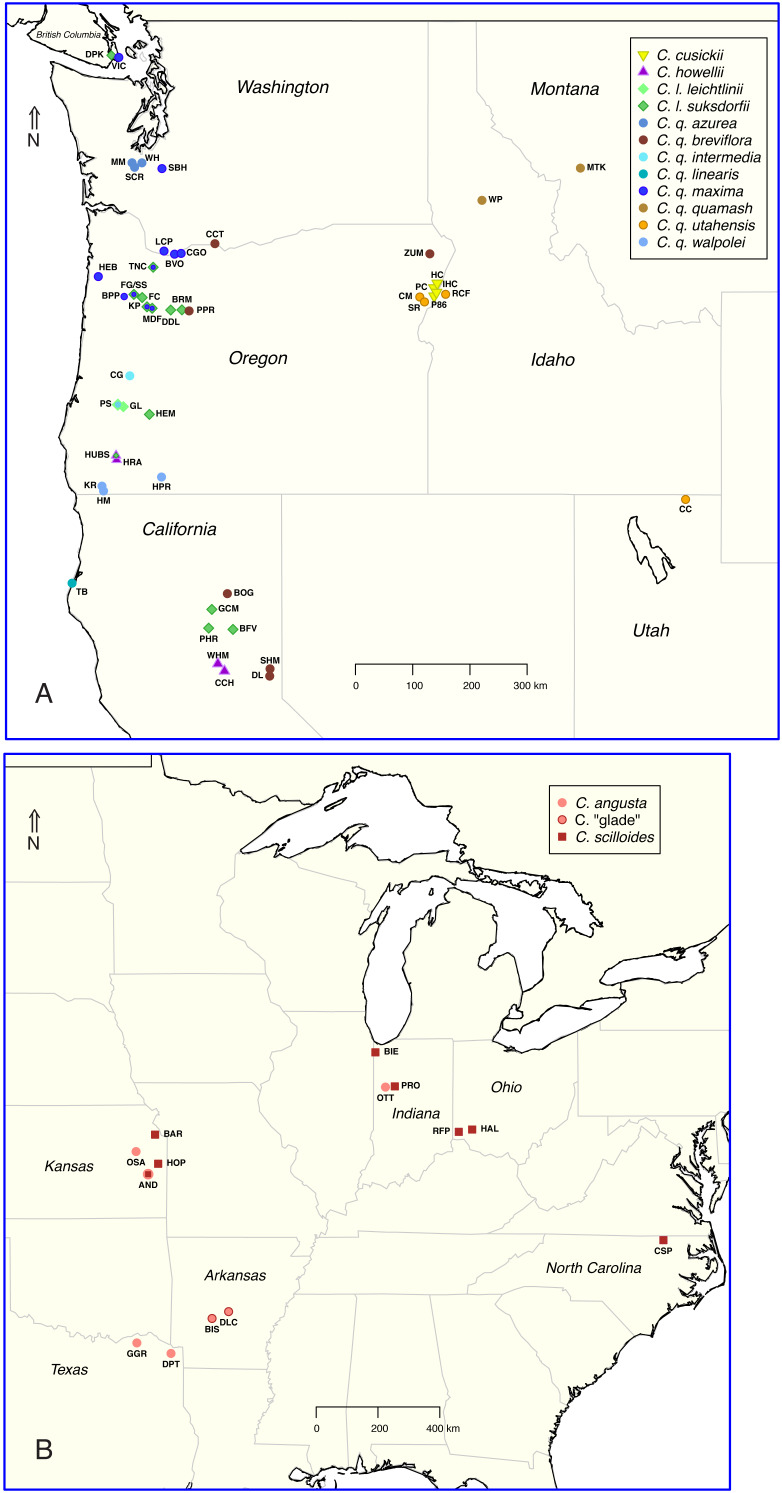
Maps showing the distribution of sampled populations of *Camassia* in North America, primarily in the (A) western and (B) midwestern to southern US.

Sampling within *Camassia* comprised 157 individuals from 71 populations, with one to five plants per population (usually two to three) and all 15 putative taxa (Appendix I, Fig. 2). These included six named species, one with eight subspecies and one with two subspecies, and one unnamed potential segregate (Table 1). Population sites were indicated by alphanumeric codes, usually with two or three characters (Appendix I). Morphologically variable, widespread taxa were sampled in more depth, with emphasis on *C. leichtlinii* and *C. quamash*, especially *C. quamash* ssp. *maxima*. We included both allopatric and sympatric populations, when possible, for species with overlapping ranges. Species and subspecies were identified in the field using author expertise, especially S. Kephart who has published extensively on this genus, including floristic treatments (Kephart, 2015; Kephart, 2018). We also used local input (see Acknowledgements) and consulted prior research and taxonomic treatments (*e.g.*, Gould, 1942; Ranker & Hogan, 2002; Ranker & Schnabel, 1986). Population boundaries were determined based on geographic distance combined with information on likely pollinator foraging distances and other barriers to gene flow (Cant et al., 2005; Danner et al., 2016). Outgroup sampling included some or all the populations collected for a separate phylogenetic study of *Hastingsia* (K Theiss et al., unpublished data, 2018) along with up to four populations from *Chlorogalum* (details below).

**Table 1:** Taxa currently described or hypothesized within *Camassia*, with their sampling for this study and full known distribution (Kephart, Kephart & Robinson, 2019; Kephart, 2015; Kephart, 2018; Ranker & Hogan, 2002).

Taxon	Number of individuals (populations) sampled	Distribution (state abbreviations for the US and province names for Canada)
*Camassia angusta* (Engelm. & A. Gray) Blank.	9 (5)	AR, IL, IN, IA, KS, MT, OK, TX
*Camassia* “glade”	3 (2)	AR
*Camassia cusickii* S. Watson	9 (4)	ID, OR, WA
*Camassia howellii* S. Watson	12 (4)	OR, CA
*Camassia leichtlinii* ssp. *leichtlinii* (Baker) S. Watson	2 (2)	OR
*Camassia leichtlinii* ssp. *suksdorfii* (Greenm.) Gould	27 (13)	British Columbia, CA, OR, WA
*Camassia quamash* ssp. *azurea* (A. Heller) Gould	6 (3)	WA
*Camassia quamash* ssp. *breviflora* Gould	16 (6)	CA, NV, OR, WA
*Camassia quamash* ssp. *intermedia* Gould	5 (2)	OR
*Camassia quamash* ssp. *linearis* Gould	3 (1)	CA
*Camassia quamash* ssp. *maxima* Gould	27 (12)	British Columbia, OR, WA
*Camassia quamash* ssp. *quamash* (Pursh) Greene	7 (2)	Alberta, British Columbia, ID, MT, OR, WA, WY
*Camassia quamash* ssp. *utahensis* Gould	7 (4)	ID, MT, OR, UT, WY
*Camassia quamash* ssp. *walpolei* (Piper) Gould	8 (3)	OR
*Camassia scilloides* (Raf.) Cory	16 (8)	Ontario, AL, AR, GA, IL, IN, IA, KS, KY, LA, MD, MI, MS, MO, OH, PA, SC, TN, TX, VA, WV, WI

Leaf material (∼20 mg or more per individual) was dried on silica gel from each population. DNA was extracted from leaf samples using standard hexadecyltrimethylammonium bromide (CTAB) methods (Doyle & Doyle, 1987) or a DNeasy Plant Mini Kit (Qiagen, Germantown, Maryland, USA). Quantity and quality of DNA were assessed using QuBit (2.0 fluorometer, Invitrogen, Waltham, Massachusetts, USA) and Nanodrop (ND-1000 Spectrophotometer, ThermoScientific, Waltham, Massachusetts, USA), respectively. Samples were chosen based on a target of 260/280 ratios between 0.9 and 2.5 and 260/230 ratios between 0.5 and 3.

### DNA sequencing

Genomic libraries were generated for 96 samples at a time, using a modified single-digest RADseq method known as multiplexed shotgun genotyping (Andolfatto et al., 2011; Baird et al., 2008). Samples were diluted to 5 ng/µL and 10 µL of template DNA was aliquoted to each well (50 ng total). A few samples with lower concentrations were diluted to 2.5 ng/µL and included in multiple wells to maximize coverage. DNA from each individual was digested with restriction enzyme AseI (NEB Biolabs, Ipswich, Massachusetts, USA), and then unique 6-bp bar-coded adapters were ligated to each sample (T4 DNA ligase, Enzymatics, Germantown, Maryland, USA). The samples were pooled and purified in separate steps using isopropanol precipitation and AMPure bead purification (Agencourt, Beckman Coulter, Brea, California, USA). Afterwards, the library was size-selected, targeting fragments of 275 bp using a Pippin Prep (Sage Science, Beverly, Massachusetts, USA), followed by another round of bead purification. Polymerase chain reaction (PCR) (14 cycles) was conducted using Phusion High-Fidelity PCR Master Mix (NEB Biolabs, Ipswich, Massachusetts, USA) and indexed primers that bind to common regions in the adaptors. PCR-products were again purified with AMPure beads, using a 0.8 × bead/template volume ratio to remove small (primer-dimer) fragments. Sequences were generated in three Illumina (San Diego, California, USA) HiSeq 2500 PE100 lanes at the Genome Sequencing Core, University of Kansas, Lawrence, Kansas, USA. Each lane typically contained up to 96 pooled individuals, including some that were sequenced for separate studies. A 10% phiX spike-in was included for all lanes to provide additional sequence complexity.

### Molecular data processing and phylogenetic analyses

We demultiplexed the FastQ files into sample-specific sequence files using custom scripts developed by John K. Kelly (Appendix S1; see Supplemental Data with this article), and processed the demultiplexed files using pyRAD *v.* 3.0.5 through ipyrad *v.* 0.7.30, using the latter for the final analyses (Eaton & Overcast, 2020). For the final sets of analyses, we ran a de novo assembly of the RADseq reads in ipyrad under the “pairgbs” datatype. We increased quality filtering stringency using filter_adapters=2 and phred_Qscore_offset=43 and employed a range of parameter settings to assess the influence on results, with particular emphasis on the influence of clust_threshold (here referred to as “c”) and min_samples_locus (here referred to as “m”). The c parameter indicates the threshold of similarity required for two loci to be identified as homologous and clustered together, and it was set at 70, 80, 90, or 95 for the final analyses. The m parameter indicates the minimum number of accessions that must have sequence data at a locus for that locus to be retained in the dataset, and it was set at 4, 8, 16, 32, or 79. For analyses of the full *Camassia* dataset (157 accessions), those m values correspond to retaining a locus found in 2.5%, 5%, 10%, 20%, and 50% of the accessions, respectively. The analysis with m79 resulted in very poorly supported trees and so was not explored further. These and a series of preliminary analyses supported c90, m04 as producing the most informative dataset in this genus and in the *Camassia–Hastingsia* clade, and so those settings were used for the rooting analyses. Due to a major observed increase in missing data when including the outgroups (see Results), we used unrooted analyses of *Camassia-*only datasets to infer relationships within the genus, while determining likely roots using multi-genera analyses. After the aligned output files were produced, we manually removed accessions with fewer than 100 loci from the datasets for all subsequent analyses.

We inferred phylogenetic relationships on concatenated alignments using maximum likelihood (ML) with the General Time Reversible (GTR)+ Γ model of nucleotide substitution in RAxML version 8.1.20–8.2.11 (Stamatakis, 2014); rapid bootstrapping analyses with 100 replicates provided relative clade support. To better account for potential gene-tree heterogeneity, we also estimated coalescent-based trees using singular value decomposition quartets (SVDquartets) (Chifman & Kubatko, 2014) as implemented in PAUP* *v.* 4a157 through 4a165 (Swofford, 2003). This method allows a coalescent-based approach, while being computationally feasible with datasets of these sizes and without estimating gene trees. Gene trees can often be unreliable when based on RADseq loci, which are relatively short. SVDquartets has also been shown to be valid with some gene flow (Long & Kubatko, 2018), which is likely to have occurred across some taxonomic boundaries in this genus (Uyeda & Kephart, 2006). We exhaustively sampled all quartets and assessed support using 100 bootstrap replicates. Preliminary analyses with 5,000 bootstrap replicates did not notably change the inferred support. We conducted individual, population, subspecies, and species tree inference under the multispecies coalescent (MSC) model. Using these two inference methods (concatenated ML and SVDquartets) allowed us to look at the combined signal of the many, relatively short RADseq loci, while also better accounting for possible deep coalescence and other factors that may cause conflict between gene trees. We also used full loci rather than Single Nucleotide Polymorphisms (SNPs), with paired-end sequencing and 100 bp reads to obtain as much information as possible per locus. Preliminary field observations suggested that *C.* “glade” may be a separate taxon. We treated it as a “species” for the purpose of the species-based tree, due to uncertainty in its status. However, if we had merged it with *C. angusta* or set it as a subspecies of *C. angusta*, its inferred placement would not have changed (see Results).

Initial analyses were run with and without one individual (*C. quamash* ssp. *maxima*_BPP_Q25). Its morphology suggested influence of gene flow from plants of *C. leichtlinii* at the same site and its inferred phylogenetic placement was far from the other individuals sampled from its population and even from other *C. quamash* (see Results and Discussion). The inclusion of this individual did not affect other inferred relationships in the individual-based phylogenetic analyses, but it did influence the population-based analyses using SVDquartets. So, we excluded *C. q. maxima*_BPP_Q25 for those analyses and most rooting analyses, leaving 156 accessions of *Camassia*.

As prior datasets have disagreed on the root of *Camassia* (Archibald et al., 2015; Fishbein et al., 2010), and given the major potential impact of outgroup choice on inferred roots (De la Torre-Bárcena et al., 2009; DeSalle, Narechania & Tessler, 2023), we compared several different rooting analyses. We also focused on reducing missing data, as there is a known tendency for increased missing data at higher phylogenetic distance with RADseq datasets (Eaton et al., 2017). The different rooting datasets used were: (1) the complete final set of all sampled accessions of *Camassia*, *Hastingsia*, and *Chlorogalum*, (2) all sampled accessions of *Camassia* (except *C. q. maxima*_BPP_Q25, discussed above) and the 20 accessions of *Hastingsia* that each shared ≥100 loci with each of ≥10 accessions of *Camassia*, (3) the accessions from *Camassia* and *Hastingsia* that each shared ≥100 loci with ≥1 accession from the other genus, and (4) all *Camassia* and *Hastingsia* accessions that each had ≥2,000 total sequenced loci (Table 2). Custom scripts developed by John K. Kelly (University of Kansas) counted the number of shared and matching loci for each pair of accessions. One additional rooting dataset included only four accessions of *Hastingsia*, focusing on those that shared a greater number of loci (≥500) with accessions of *Camassia*. However, results from those analyses were highly incongruent with all others, including nesting one or more individuals of *Hastingsia* deep in the *Camassia* clade. A close examination of those results suggested it was a spurious result of missing data, and so they will not be discussed further.

**Table 2:** Characteristics of RADseq datasets of *Camassia* and outgroups from *Hastingsia* and *Chlorogalum*.

Dataset name	No. individuals	c^a^	m^b^	Percent of full accession set covered by m	Total no. loci in the dataset^c^	Average no. loci per accession^d^	No. variable characters^e^	No. parsimony informative sites^f^
*Camassia*	157	70	4	2.5	28,177	1,737	305,883	107,076
	157	80	4	2.5	47,067	2,679	522,363	177,852
	157	90	4	2.5	103,800	5,816	1,174,784	400,398
	157	95	4	2.5	137,433	7,414	1,177,519	365,761
	157	80	8^g^	5.1	17,417	1,744	269,771	111,222
	157	90	8	5.1	38,804	3,747	623,682	253,182
	157	95	8^g^	5.1	49,395	4,602	642,019	228,509
	157	80	16^g^	10.2	5,735	974	117,930	51,674
	157	90	16	10.2	12,077	1,980	255,939	112,015
	157	95	16^g^	10.2	14,464	2,288	262,215	100,418
	157	90	32	20.4	2,550	697	66,926	30,722
	157	90	79	50.3	47	26	1,324	631
Rooting 1^h^	198	90	4	2.0	143,404	6,214	1,647,614	600,538
Rooting 2^i^	176	90	4	2.3	133,894	6,492	1,526,078	547,161
Rooting 3^j^	110	90	4	3.6	118,890	9,222	1,370,438	492,810
Rooting 4^k^	147	90	4	2.7	135,271	7,988	1,544,900	565,870

**Notes.**

a c = similarity threshold for clustering loci as homologous in the de novo assembly.

b m = minimum number of accessions that must have sequences for a given locus for that locus to be included in the dataset.

c From ipyrad’s “total_filtered_loci”.

d Calculated from ipyrad’s “sample_coverage”.

e From ipyrad’s “sum_var”.

f From ipyrad’s “sum_pis”.

g Phylogenetic analyses were not conducted on the indicated datasets.

h Rooting 1 dataset: all sequenced *Camassia* (157 accessions), *Hastingsia* (36), and *Chlorogalum* (5).

i Rooting 2 dataset: all sequenced *Camassia* (except *C. quamash* ssp. *maxima*_BPP_Q25; 156 accessions) and the 20 accessions of *Hastingsia* that each shared ≥100 loci with ≥10 accessions of *Camassia*.

j Rooting 3 dataset: the 90 accessions of *Camassia* and 20 of *Hastingsia* that each shared ≥100 loci with at least one accession of the other genus.

k Rooting 4 dataset: the 118 accessions of *Camassia* and 29 of *Hastingsia* that each were sequenced for ≥2,000 total inferred loci.

## Results

### RADseq loci across and within the genera, and rooting of *Camassia*

**Figure 3:**
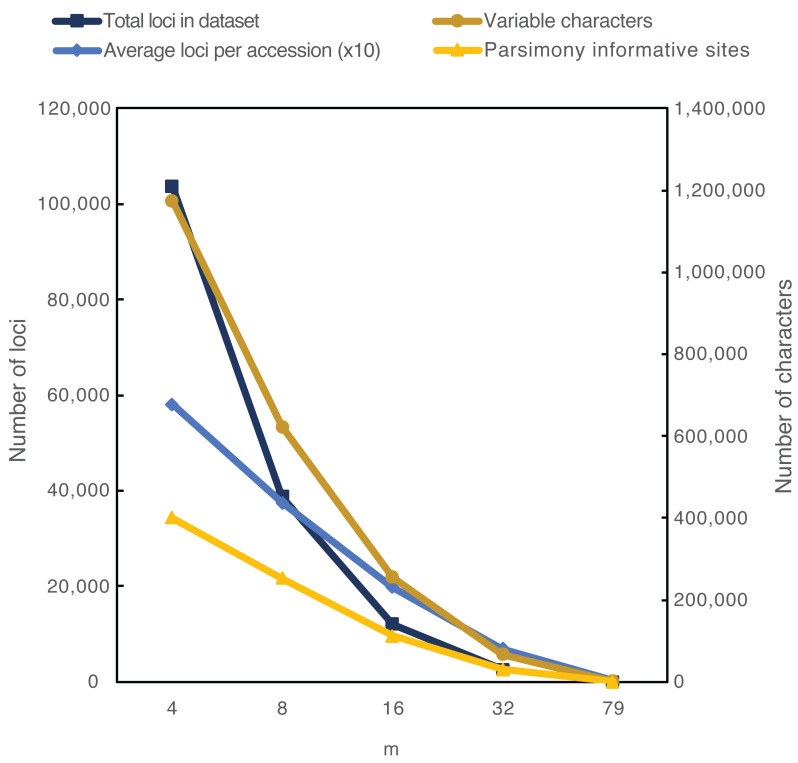
Comparisons of datasets for 157 accessions of *Camassia* with ipyrad parameters of c90 and the indicated values of m. The m parameter indicates the minimum number of accessions that must have sequence data at a locus for that locus to be retained in the dataset. The average numbers of loci per accession have each been multiplied by 10 to allow clearer visualization.

The average sequencing depth for loci from each accession ranged from 10 to 117 across *Camassia, Hastingsia*, and *Chlorogalum*, while the average number of usable reads per accession was 21,212 (reads_consens in ipyrad). Within the focal 157 accessions of *Camassia*, the overall average depth was 33 and that did not change with varying ipyrad parameters. The number of loci within the *Camassia* datasets varied from over 137,433 to only 47, dropping with higher m values (Table 2, Fig. 3). When we used m32 as our largest m value (excluding m79), the smallest number of total loci was 2,550. The average number of loci per individual was much lower than the total number of loci across individuals, demonstrating the lack of overlap of many loci across individuals and a correspondingly high level of missing data. The number of parsimony informative sites showed a similar trend, ranging from over 400,398 to 631 depending on the parameters (with a minimum of 30,722 if excluding the m79 analyses).

Decreasing missing data by excluding more loci resulted in lower clade support. With the m parameter at 04 (including a locus in the dataset even if only 2.5% of the accessions had that locus), ML and SVD analyses resolved 100 and 58 clades with >90% bootstrap (BSt) within *Camassia*, respectively. In contrast, analyses of the same accessions with m32 (requiring 20.4% accessions) resolved less than half that number of strongly supported clades (46 and 26 clades). Less taxonomic cohesion also was evident at higher m values. For example, nine putative taxa were resolved as monophyletic with SVD analyses of the m04 individual dataset but only five with m32. At the population level, there was a 38% and 18% decrease in strongly supported monophyletic populations for ML and SVD analyses, respectively, when changing from m04 to m32. The effect of sacrificing signal to avoid missing data was even more clear if we required loci to be found in at least 50% of the accessions (m79 for the *Camassia* dataset); only 47 loci remained in that dataset and there were almost no strongly supported clades in the resulting trees.

Many more loci were shared within *Camassia* than between it and the outgroup genera. On average, pairs of accessions of *Camassia* shared 252 loci (based on 12,246 pairs of accessions), whereas *Camassia*–*Hastingsia* pairs shared 53 loci (5,652 pairs). Regardless, all relationships among the genera had bootstrap support of 100%. The overall levels of support and relationships inferred within *Camassia* were similar when loci were processed and analyzed separately for this genus or alongside those for the other two genera, despite the steep increase in missing data. That consistency was seen for both deep and shallow relationships within the genus. One well-supported exception was the ML placement of the *C. cusickii*+ clade, where the ML analyses of datasets with multiple genera moved the *C. cusickii*+ clade into the *C. quamash* clade, sister to the *C. q. breviflora*+ clade (BSt = 100%).

**Figure 4:**
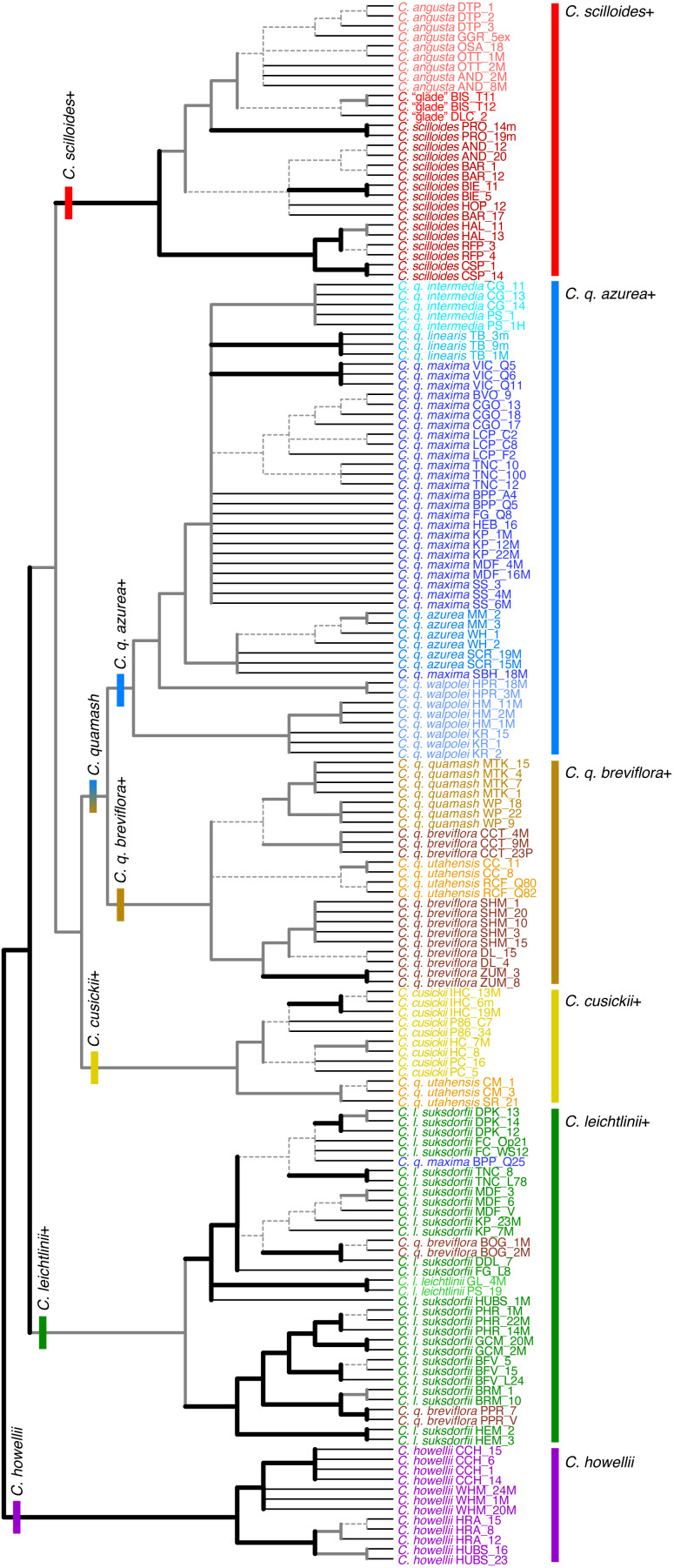
Individual-level phylogenetic relationships inferred using RADseq data from *Camassia*. Relationships are summarized for 10 analyses conducted in both RAxML and SVDquartets with these ipyrad settings: c80m04, c90m04, c90m08, c90m16, c95m04. Regardless of parameter settings (with few exceptions), thick black branches were resolved and strongly supported (>90% Bootstrap, BSt)^*a*^, and thick gray branches were resolved but with variable levels of support^*b*^. Dashed branches varied in resolution and support but show the relationships inferred and most strongly supported by the majority of analyses. Inferred relationships were collapsed into polytomies if there was no dominant pattern supported across analyses with different parameters. Thin black branches lead to tips. Major clades are indicated with bars at their base and to the right of taxon names. These analyses were unrooted; the tree shows one of two possible roots supported by separate rooting analyses. The alternative resulted in a *C. howellii*–*C. leichtlinii*+ clade as sister to the rest of *Camassia*. See text for details. ^*a*^If a relationship had >90% BSt for all analyses except for one with >75% BSt, the line was left as thick black. ^*b*^If a relationship was resolved by all analyses except for one, and the conflicting relationship had <50% BSt support, the line was left as thick gray.

**Figure 5:**
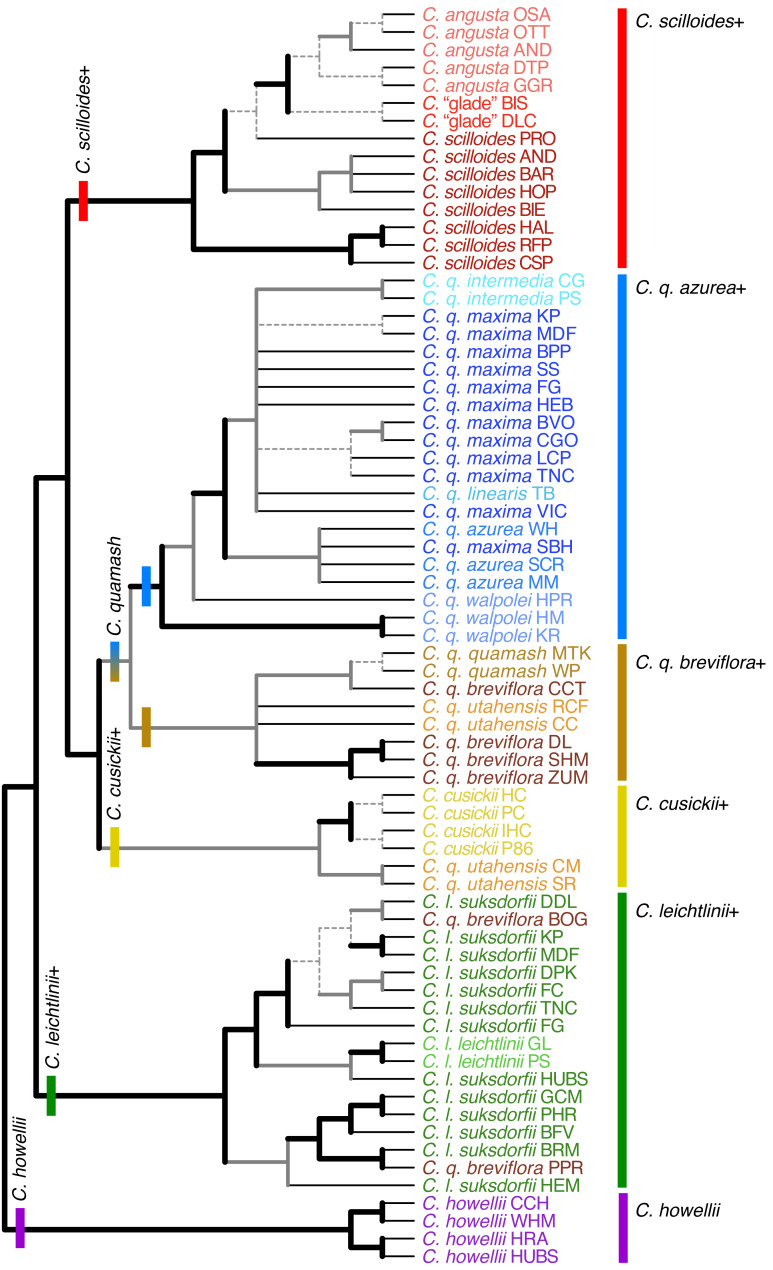
Population-level phylogenetic relationships inferred using RADseq data from *Camassia*. Relationships are summarized for five analyses conducted in SVDquartets with these ipyrad settings: c80m04, c90m04, c90m08, c90m16, c95m04. Regardless of parameter settings (without exceptions), thick black branches were resolved and strongly supported (>90% bootstrap, BSt), and thick gray branches were resolved but with variable levels of support. Dashed branches varied in resolution and support but show the relationships inferred and most strongly supported by the majority of analyses. Inferred relationships were collapsed into polytomies if there was no dominant pattern supported across analyses with different parameters. Thin black branches lead to tips. Major clades are indicated with bars at their base and to the right of taxon names. These analyses were unrooted; all separate population-level rooting analyses supported the root shown in the figure.

**Figure 6:**
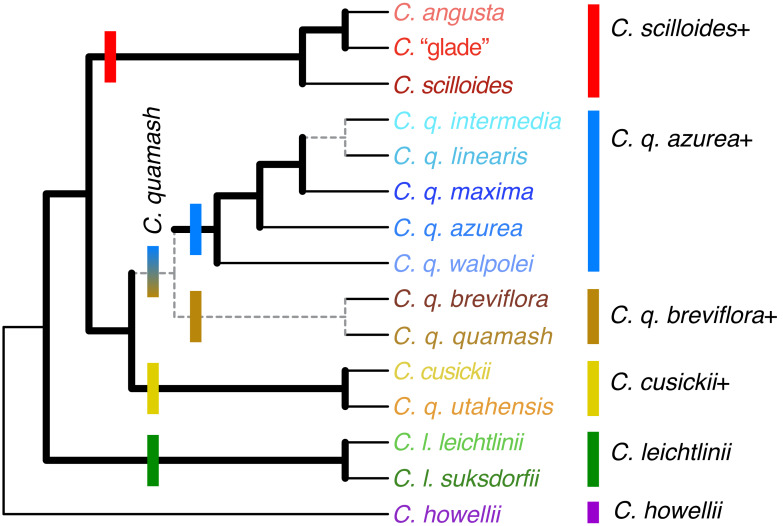
Subspecies-level phylogenetic relationships inferred using RADseq data from *Camassia*. Relationships are summarized for five analyses conducted in SVDquartets with these ipyrad settings: c70m04, c90m04, c90m16, c90m32, c95m04. Regardless of parameter settings (without exceptions), thick black branches were consistently resolved and strongly supported (>90% bootstrap, BSt); all those branches had bootstrap values of 100%, excepting a few from the c70m04 analysis. Dashed branches varied in resolution and support but show the relationships inferred and most strongly supported by the majority of analyses. Thin black branches lead to tips. Major clades are indicated with bars at their base and to the right of taxon names. These analyses were unrooted; all separate subspecies-level rooting analyses supported the root shown in the figure.

**Figure 7:**
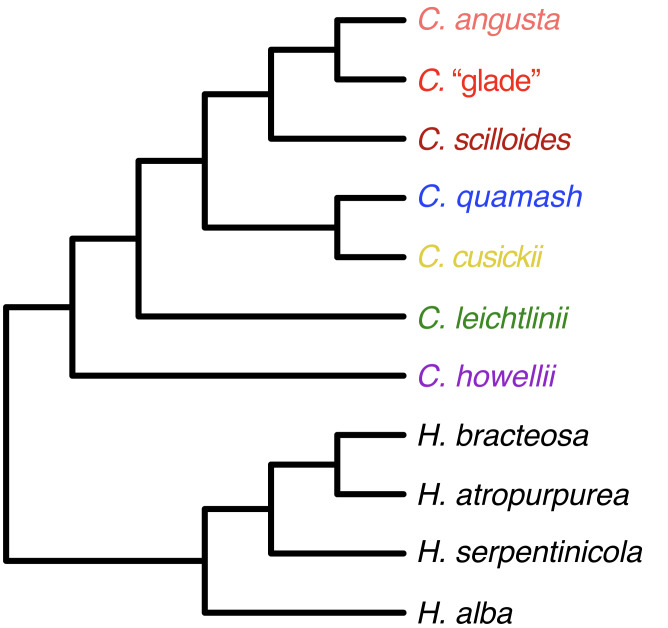
Species tree relationships inferred using RADseq data from *Camassia* and *Hastingsia*. Relationships are summarized for five analyses conducted in SVDquartets of *Camassia* accessions alone (using ipyrad parameters c70m04, c90m04, c90m16, c90m32, and c95m04) and four of *Camassia* and *Hastingsia* (using c90m04). All relationships had 100% bootstrap support in all analyses, except for the *H. bracteosa*–*H. atropurpurea* clade in one analysis (97% BSt) and the sister relationship between *C. howellii* and the rest of *Camassia* in another (99% BSt).

The basal relationships in *Camassia* were inferred as one of two possibilities by rooting analyses. Either *C. howellii*–*leichtlinii* was sister to the rest of *Camassia*, or *C. howellii* and *C. leichtlinii* formed a grade at the base of *Camassia*, with *C. howellii* sister to the rest of *Camassia*. Maximum likelihood analyses supported the former, whereas SVD analyses supported the latter. We chose the *C. howellii* root when illustrating relationships (Figs. 4–7), given that this root was supported at all taxonomic grouping levels and by taxonomically broader analyses using data from nuclear and chloroplast loci (Archibald et al., 2015). Not illustrated, but possibly of interest to some readers are the relationships among our few sampled *Chlorogalum* accessions: two populations of *C. pomeridianum* (DC.) Kunth var. *pomeridianum* formed a strongly supported clade sister to *C. p.* var. *minus* Hoover (with variable support), and this clade was sister to two individuals from a population of *C. p.* var. *divaricatum* (Lindl.) Hoover. This differed from Archibald et al. (2015), which instead supported *C. p.* var. *pomeridianum* as sister to *C. p.* var. *divaricatum*.

### Relationships within *Camassia*

#### Consistency across parameter sets

Representing analyses run using different parameter sets, different levels of grouping, and both ML and SVD optimization, our tree figures summarize phylogenetic relationships inferred across 25 different analyses of the *Camassia*-only datasets (Figs. 4–7). Our tree of species-level relationships also summarizes four of the rooting analyses of *Camassia* and *Hastingsia* (Fig. 7). Overall, the deeper relationships in *Camassia* were fully resolved and well supported, whereas some relationships within species were not strongly supported.

Analyses using RAxML and SVD were largely consistent; the most common difference was that some relationships were not as strongly supported in SVD analyses as in RAxML. All inferred relationships were consistent across SVD analyses with different grouping of individuals (*i.e.,* without grouping, grouping as populations, as subspecies, and as species). In this case, “consistent” means that most inferred clades were identical, with occasional minor differences where a relationship was partially supported by one set of analyses but unresolved by another. For example, partial support was found in cases where some analyses in a set resolved a given relationship, and others did not (indicated by dashed branches in the trees). In other cases, the relationship was inferred by all analyses in the set but with variable support (indicated by solid gray branches in the trees). However, even the relationships indicated by dashed branches were often identically resolved by the individual, population, subspecies, and species analyses. Some populations or taxa were not completely monophyletic in individual-based analyses. When forced to be monophyletic by grouped SVD analyses, the placement of those lineages was unsurprising, following the majority of their accessions. Specific cases are discussed below.

#### General patterns of relationships

Each of the species of *Camassia* was supported as fully monophyletic, monophyletic excepting a few outliers, or paraphyletic. *Camassia howellii*, *C. cusickii*, and *C. angusta* s.l. (*i.e.,* if encompassing *C.* “glade”) were each monophyletic in all analyses. *Camassia scilloides* formed a grade at the base of a strongly supported clade of *C. angusta* and *C.* “glade”. The 79 individuals sampled from 34 populations of the 8 subspecies of *C. quamash* formed a largely monophyletic group, except for a few populations that moved out and disrupted the otherwise monophyletic *C. leichtlinii* (Figs. 4 and 5).

Within *C. leichtlinii*, *C. l. leichtlinii* was monophyletic but nested within clades of *C. l. suksdorfii* (Greenm.) Gould. Even with broad sampling of 29 accessions from 15 populations of this species, many relationships were strongly and consistently supported across all analyses. The species was divided into two strongly supported clades and many populations were supported as monophyletic (none were contradicted, but some were unresolved). Relationships within *C. leichtlinii* were identical when comparing the individual-level and population-level phylogenies, except that the *C. l. leichtlinii* clade and *C. l. suksdorfii*_HUBS formed a polytomy with the rest of the members of one of the two subclades of *C. leichtlinii* in individual-level analyses (Fig. 4) and were a well-supported sister clade to the rest of that subclade in population-level analyses (Fig. 5). The *C. leichtlinii+* clade included phylogenetic outliers from three populations of *C. quamash*: two populations of *C. q. breviflora* (BOG and PPR) and one individual of *C. q. maxima* (population BPP).

The main *C. quamash* clade was divided into two well-supported clades: the *C. q. azurea*+ clade and the *C. q. breviflora*+ clade (Figs. 4–6). Each subspecies of *C. quamash* was restricted to one of these two main clades, with a few exceptions (see below). The *C. q. azurea*+ clade included a well-supported subclade of *C. q. intermedia*, *C. q. linearis*, and *C. q. maxima*, sister to *C. q. azurea*, with *C. q. walpolei* forming a grade at the base. Two narrowly distributed taxa were each well supported as monophyletic: *C. q. intermedia* (two populations, five individuals sampled) and *C. q. linearis* (one population, three individuals). In contrast, relationships among the 27 individuals (12 populations) of wide-ranging *C. q. maxima* were generally not well resolved beyond forming a clade with *C. q. intermedia* and *C. q. linearis*, with two outliers. One population of *C. q. maxima* (SBH, with one individual sampled) was placed in the otherwise-monophyletic *C. q. azurea* clade rather than with other members of its subspecies. The other outlier (*C. q. maxima* _BPP_Q25) fell farther, in the *C. leichtlinii* clade. However, other sequenced individuals from this population were placed with the main group of *C. q. maxima* individuals. As noted in the Methods, *C. q. maxima*_BPP_Q25 was removed from some analyses due to possible influence of gene flow. Forming a grade at the base of the *C. q. azurea*+ clade, two populations of *C. q. walpolei* (HM, KR) formed a clade, whereas population HPR was sister to a large clade containing the other subspecies of the *C. q. azurea*+ clade.

The *C. q. breviflora*+ clade included *C. q. breviflora*, *C. q. quamash*, and two of four populations of *C. q. utahensis.* The monophyly of *C. q. quamash* had mixed support, with stronger support from individual-based analyses than some population-based analyses. None of the analyses resulted in a monophyletic *C. q. breviflora*. One population of *C. q. breviflora* (CCT) was instead usually inferred as sister to *C. q. quamash*, although the support for this division was weak. Two populations of *C. q. breviflora* (BOG and PPR, two individuals sampled for each) were each monophyletic and strongly supported as members of different subclades within the *C. leichtlinii*+ clade in both the individual and population-based trees. When *C. q. breviflora* was forced to be monophyletic by subspecies-level SVD analyses, the lineage remained with the rest of *C. quamash*. Finally, *C. q. utahensis* was divided by individual- and population-level analyses, with two populations (CC and RCF) in the *C. q. breviflora*+ clade and two populations (CM and SR) placed sister to *C. cusickii*. This *C. cusickii*–*C. q. utahensis*_CM,SR clade was then sister to the entire *C. quamash* clade. In the five subspecies-level SVD analyses, *C. q. utahensis* was forced to be monophyletic and was sister to *C. cusickii* (BSt ≥ 94% for all) rather than with the remainder of its species, but there was either reduced support for the monophyly of the remainder of *C. quamash* (three analyses: c90m04, c90m16, c90m32) or that monophyly was disrupted by the movement of the *C. cusickii*–*C. q. utahensis* clade to be nested with other members of the *C. q. breviflora*+ clade (two analyses: c70m04 and c95m04, also found in some rooting analyses, as noted above). In both cases, forcing monophyly of *C. q. utahensis* lowered support for some containing clades of that subspecies compared to analyses where populations were able to move separately within the phylogeny.

The more eastern clade of *Camassia* had the most notable difference between RAxML and SVD individual-based analyses. Some SVD analyses intermixed populations of *C.* “glade” and *C. angusta*, whereas those two groups were strongly supported as sister and reciprocally monophyletic by RAxML. Regardless, together *C. angusta* and *C.* “glade” formed a well-supported clade (Figs. 4–7).

## Discussion

### RADseq utility and missing data

We found general utility of RADseq for inferring relationships across recently diverged genera, species, and subspecies in this group, although not all relationships within subspecies were clear. These datasets were comprised of over 137,000 loci within *Camassia*, with over 400,000 parsimony informative sites. As seen elsewhere, the parameters used for filtering and determining homology of loci greatly influenced their numbers (Leaché et al., 2015; Takahashi, Nagata & Sota, 2014; Wessinger et al., 2016), and signal remained even with a large proportion of missing data (Crotti et al., 2019; Huang & Knowles, 2016; Lee et al., 2018). Fewer loci in RADseq datasets with higher m values was expected, although the loss of signal was more severe here than in some studies. For example, several studies generally focused on clades ranging in age from 2 to 16 myo (Appelhans et al., 2018; Maier et al., 2019; Sharples & Manzitto-Tripp, 2024) successfully used m values that included a locus if 13–60% of the accessions had sequences for that locus (Maier et al., 2019; Paetzold et al., 2019; Raheem et al., 2023; Sharples & Tripp, 2019). Comparing to *Camassia* at approximately 5.22 myo (Smith et al., 2008), setting m to require even as much as 20% sampling resulted in lowered support for many clades when compared to analyses with more loci (and more missing data). Insufficient sequencing effort can be a source of missing data in RADseq datasets (Eaton et al., 2017), but that was likely not a problem here, given our high sequencing depth (Andrews et al., 2016). We also saw decreases in the number of supported monophyletic taxa and populations when excluding loci to lower the amount of missing data. A taxon or population being non-monophyletic could accurately reflect the biology of that group. However, multiple identifiable taxa or populations being resolved as monophyletic in one set of analyses and not monophyletic in another set suggests loss of signal in the latter. The pull of *C. cusickii*+ towards the *C. q. breviflora*+ clade, seen primarily in some three-genus analyses, highlights one potential impact of missing data. This alternate placement may relate to an affinity between a member of the latter clade (*C. q. utahensis*) and *C. cusickii* (discussed below), and it was also inferred by two subspecies-level analyses where *C. q. utahensis* was forced to remain monophyletic. Regardless, and despite high levels of missing data, our datasets allowed resolution of consistent and well-supported phylogenetic hypotheses across a range of parameters for many relationships (Figs. 4–7).

### The root and early diversification within *Camassia*

The monophyly of *Camassia* was very strongly supported across all multi-genera analyses (100% BSt). Our analyses highlighted the importance of careful rooting. Different analyses strongly supported different roots despite choosing outgroups from the closest relatives of this genus and choosing accessions to maximize signal. We found support for two likely roots for *Camassia*, either a *C. howellii–C. leichtlinii*+ clade (supported by ML analyses) or *C. howellii* alone, with *C. leichtlinii*+ then being sister to the remainder of *Camassia* (supported by SVD analyses at all grouping levels). In prior analyses using Bayesian and maximum parsimony, Fishbein et al. (2010) weakly supported *C. howellii–C. leichtlinii*+ as sister to the rest of *Camassia* using two chloroplast loci, while the combined ITS and cpDNA analyses of Archibald et al. (2015) found strong support for *C. howellii* as sister. Our data strongly reinforce those two hypotheses. In either scenario, we saw that the *C. howellii* and *C. leichtlinii*+ clades split early from the remainder of *Camassia* and the unrooted basal relationships within *Camassia* were identical and strongly supported across most analyses. Consistent with these conclusions, Gould (1942) proposed an early origin of both *C. howellii* and *C. leichtlinii*, especially *C. howellii*. This was based on its morphological similarity to related genera, particularly *Chlorogalum*. In the next section, we focus within *C. leichtlinii*, while Kephart et al. (2025) separately discuss integrative studies of *C. howellii* and its relationships with sympatric species, using morphology, microsatellite, and cpDNA data.

### Geographic patterns and gene flow in the well-resolved and widespread *Camassia leichtlinii*

*Camassia leichtlinii* extends from the northern half of California in the US, northward into British Columbia in Canada and consists of two subspecies, of which, *C. l. leichtlinii* is native to just one county in Oregon (Fig. 2; Ranker & Hogan, 2002). In Oregon and northward, *C. l. suksdorfii* occurs from the coast to the slopes of the Cascade mountains, but rarely east of the Cascades (Fig. 1 in Gould, 1942), and it is often sympatric with *C. quamash*. In California, *C. l. suksdorfii* populations grow on both sides of the Cascades and in the Sierra Nevada mountains, and it has not been found in sympatry with *C. quamash* (data provided by the participants of the Consortium of California Herbaria, ucjeps.berkeley.edu/consortium/).

The subspecies differ in flower color, creamy-white in *C. l. lecihtlinii* and variable blue to deep violet, rarely white, in *C. l. suksdorfii* (Figs. 1A, 1B; Gould, 1942). Large, diurnal flowers, five to nine tepal veins, and oblong fruits separate *C. leichtlinii* from its potential sister species *C. howellii*, which has smaller, vespertine flowers, three to five tepal veins, and subglobose fruits (Kephart, 2015). Comparing western species in the genus, flowers of *C. howellii* and *C. leichtlinii* abscise at the pedicel tip if no fruit is matured, while *C. cusickii* and *C. quamash* retain spent flowers on intact pedicels (Kephart, 2015). Although *C. leichtlinii* is distinctive in morphology, habitat differentiation is less obvious. This species, like *C. quamash*, inhabits varied wet meadow, riparian, and oak savannah communities in diverse soil types from near sea-level to 2,400 m (Ranker & Hogan, 2002; Sultany, Kephart & Eilers, 2007). However, when in sympatry with *C. quamash* and *C. howellii*, *C. leichtlinii* prevails in partial shade or wetter microhabitats of open sites, rather than the more open areas where the other two species are found (Sultany, Kephart & Eilers, 2007).

The monophyly of *C. leichtlinii* was well-supported across our analyses (Figs. 4–6), with the addition of a few outliers from *C. quamash*, discussed below. This species had the strongest internal support across the phylogenies, with two main clades (Figs. 4 and 5). One clade included relatively high elevation populations (ranging from around 1,100 to 1,670 m) and the other held low elevation populations (from around 50 to 460 m). This division is largely consistent with relationships inferred previously (Archibald et al., 2015; Fishbein et al., 2010), although two populations that were sampled by both studies (HUBS and BFV) were placed differently by analyses of Fishbein et al. (2010), *C. l. suksdorfii*_BFV was sister to the rest of *C. leichtlinii*+ and *C. l. suksdorfii*_HUBS was nested within *C. howellii*.

Considering our two clades within *C. leichtlinii*+, the montane (high-elevation) clade included five populations of *C. l. suksdorfii*, along with outlier *C. q. breviflora*_PPR. Relationships within this clade mirrored geography. A basal grade of Oregon Cascade populations (*C. l. suksdorfii*_HEM, BRM, and *C. q. breviflora*_PPR) fell with a nested subclade of three California populations (a subclade of *C. l. suksdorfii*_GCM–PHR from the Cascades, sister to BFV from the Sierra Nevadas). Our clade of low-elevation populations included 10 populations from both subspecies of *C. leichtlinii* and two populations of *C. quamash*. All 12 occur below 460 m, excepting one of the two outlier populations (*C. q. breviflora*_BOG at 1,889 m). Within this clade, some relationships reflected geographic patterns and others did not. The *C. l. leichtlinii* clade and *C. l. suksdorfii*_HUBS have a potentially close relationship (Fig. 5) and are also only ∼80 km apart geographically, compared to ∼170 to 690 km distance between the *C. l. leichtlinii* clade and any other population in this subclade. These three sites also lie along a well-traveled route today and historically for indigenous tribes and European settlers (Reinhardt, 2020), providing a strong possibility of human-mediated migration (Auffret, Berg & Cousins, 2014; Silcock et al., 2024). In contrast, a lack of geographic correspondence was seen with *C. l. suksdorfii*_DDL, which lies just 25 km from *C. l. suksdorfii*_BRM, but was placed in a separate subclade.

Given recognized hybridization with *C. quamash* (Uyeda & Kephart, 2006), it may not be surprising that a few individuals of putative *C. quamash* were resolved phylogenetically within the *C. leichtlinii*+ clade. However, we also saw support for the general integrity of these species’ boundaries, such as at the sympatric site PS, where sequenced individuals of *C. l. leichtlinii* and *C. q. intermedia* were each placed with their respective species in the phylogeny (Fig. 4). Overall, the *C. leichtlinii*+ clade is no more closely related to the *C. quamash* clade than it is to any other species of *Camassia* (Figs. 4–7). Considering current geography and opportunity for gene flow, outliers were strongly supported as members of both major subclades of *C. leichtlinii*, but all populations of *C. leichtlinii* in the high-elevation clade are allopatric with other species of *Camassia*, while most in the low-elevation subclade are sympatric with or grow within 2–5 km of *C. q. maxima*. As noted above, placement of outlier *C. q. maxima*_BPP_Q25 is most plausibly due to local introgression; other sampled individuals from this population lie within the *C. quamash* clade with their subspecies. At this site, *C. quamash* typically flowers 2–3 weeks before *C. l. suksdorfii*, limiting hybridization (Uyeda & Kephart, 2006). However, some likely hybrids have been collected, and this individual (Q25) was noted as having morphologically intermediate traits while appearing closer to the known morphology of *C. q. maxima* (S Kephart, pers. obs., 2010). The outlier individuals of *C. q. breviflora* are from populations BOG and PPR. Accession V of *C. q. breviflora*_PPR was also sampled by Fishbein et al. (2010) and resolved within a *C. leichtlinii*+ clade using chloroplast loci; its placement was ascribed to ancestral polymorphism or prior gene flow. Those authors excluded recent chloroplast capture, largely because the outlier fell in a subclade of geographically distant populations of *C.  leichtlinii*. However, our results show *C. q. breviflora*_PPR as sister to a population not sampled by Fishbein et al. (2010), *C. l. suksdorfii*_BRM, which occurs within ∼4 km of this outlier. A separate, integrative study on *Camassia* populations in the California Floristic Province includes *C. q. breviflora*_BOG and allows a closer look at the morphology and phenology of this population compared to *C. leichtlinii* (Kephart et al., 2025). Similarly, population-level studies of *C. q. breviflora*_PPR, with sampling of additional individuals from this population and nearby populations of *C. leichtlinii* would be useful. This would be an additional check against potential misidentification or contamination of samples–although the likelihood of that occurring is low given careful specimen handling and detailed morphological examination to identify individuals. Regardless, deeper sampling would allow a more detailed view of genetic and morphological diversity, within and between the two species.

### The many subspecies of *C. quamash* and a possibly porous species boundary with *C. cusickii*

The taxonomically and morphologically diverse *C. quamash* was subdivided into two well-supported clades (Figs. 4–6). Members of the *C. q. azurea*+ clade occur west of the Cascade mountains in the Pacific Northwest region of the United States, with a moderate climate and abundant rainfall; members of the *C. q. breviflora*+ clade occur in the rain shadow east of the Cascades within dry semi-arid and high desert areas with diurnal and seasonal temperature extremes (Fig. 2; Ranker & Hogan, 2002; Taylor & Hannah, 1999). We will discuss each subspecies within these two clades, with hypotheses for unexpected phylogenetic relationships, how they may connect to geographic patterns, and their taxonomic implications. Morphological traits have clearly diverged across these subspecies, but not always with sharp or universal distinctions among them.

### Rethinking the subspecies of *C. quamash* west of the Cascades (the *C. q. azurea*+ clade)

Despite field, herbarium, and phylogenetic studies (Archibald et al., 2015; Fishbein et al., 2010; Gould, 1942), taxon boundaries have remained uncertain among the five westernmost subspecies of *C. quamash*, including *C. q. walpolei*, *C. q. azurea*, *C. q. intermedia*, *C. q. linearis*, and *C. q. maxima*. Possibly the most morphologically distinctive is *C. q. walpolei*, a subspecies known from southwest Oregon in the Siskiyou-Klamath region, growing in wet meadows or forest openings (Kephart, 2015). In his monograph of *Camassia*, Gould (1942) referred to *C. q. walpolei* as “the best defined subspecies of *C. quamash*”. It is morphologically distinct from others in the *C. q. azurea*+ clade in its primarily radial rather than zygomorphic symmetry, smaller flowers, and fruits with shorter pedicels and fewer seeds (Ranker & Hogan, 2002). This subspecies also differs from others in this clade in that ∼74% of sampled plants have tepals with three veins instead of five to nine (Gould, 1942; Kephart, 2015). The only subclade of *C. q. azurea*+ that was well supported by prior analyses comprised two populations of *C. q. walpolei* (Archibald et al., 2015); our increased population sampling revealed paraphyly of this subspecies, which formed a grade at the base of the *C. q. azurea*+ clade (Figs. 4 and 5). Although morphological traits can vary developmentally or among populations within subspecies of *C. quamash* (Kephart, 2015), *C. q. walpolei* is distinctive in its morphology and phylogenetic relationships, suggesting that it be maintained taxonomically.

Like *C. q. walpolei*, *C. q. azurea* has a limited range, but it grows on grassy mounds or in prairies of western Washington. Most prevalent in glacial outwash soils of counties near South Puget Sound and the Olympic Peninsula, *C. q. azurea* ranges as far north as Whidbey Island (Kephart, 2018). Only *C. q. maxima* comes close geographically to *C. q. azurea*, but *C. q. maxima* grows in wet meadows to rocky bluffs at unglaciated sites that are largely allopatric to *C. q. azurea* (Gould, 1942). Morphologically, several traits distinguish *C. q. azurea* from the other subspecies. In Gould’s key, the pale blue-violet perianths of *C. q. azurea* differ from the deeper blue-violet of *C. q. maxima*, although he notes intergradation in floral color and bulb traits of these subspecies in Washington. Spreading *vs.* appressed fruit pedicels separates *C. q. azurea* (spreading in ≥ 95% of individuals) from six of the remaining seven subspecies (fruits appressed to stems in ≥ 99% of individuals), while *C. q. maxima* is variable (60% of fruits appressed; Gould, 1942). Initial morphometric data reveal potential differences between *C. q. azurea* and *C. q. maxima* in bract length relative to pedicel length, but also variable floral color in *C. q. maxima*; thus, wider sampling is essential (Theiss & Kephart, 2015). An ecological difference between *C. q. azurea* and *C. q. maxima* is seen in attacks of cecidomyiid flies (*Dasineura camassiae*) that induce camas to form flower galls, with putative host specificity (Barosh & Kephart, pers. obs., 2012; Gagné, Barosh & Kephart, 2014). Galls occurred in over half of populations of *C. q. azurea* that we observed but have not been found in any populations of *C. q. maxima* (N >18), even at sites that house other gall-forming *Camassia* taxa (Barosh & Kephart, pers. obs., 2012).

Our RADseq analyses provided the first phylogenetic evidence for a potentially monophyletic *C. q. azurea* (Figs. 4 and 5); it was previously within a polytomy (Archibald et al., 2015; Fishbein et al., 2010). Only our single individual from *C. q. maxima*_SBH falls within the otherwise-monophyletic *C. q. azurea*. Due to geographic proximity, prior gene flow from *C. q. azurea* to *C. q. maxima*_SBH is plausible *via* pollen transport by bees or seed dispersal from floristically similar glacial outwash prairies in Thurston County (Kruckeberg, 1991). For example, glacial meltwater may have connected *C. q. azurea*_WH and *C. q. maxima*_SBH (separated by ∼32 km). It is yet unknown whether these two subspecies can cross-fertilize. In all, while questions remain about these variable taxa, our data support some distinctiveness of *C. q. azurea*, thus we do not recommend taxonomic changes with this subspecies at present.

The remaining subspecies in the *C. q. azurea+* clade formed a well-supported subclade in the RADseq phylogenies, comprising *C. q. intermedia*, *C. q. linearis*, and *C. q. maxima* (Figs. 4–6). The ranges of *C. q. intermedia* and *C. q. linearis* are relatively narrow and fully disjunct from each other, in southwestern Oregon and northwestern California, respectively (Ranker & Hogan, 2002). Despite its limited range in California, *C. q. linearis* occurs in diverse sites, representing north coastal bluffs, wetlands, and montane slopes of the coast range (K Theiss, pers. obs., 2024; Gould, 1942). In contrast, strictly inland *C. q. intermedia* grows in open or wooded wet areas (Kephart, 2015). Broader-ranging *C. q. maxima*, in Oregon, Washington, and British Columbia, is also disjunct from *C. q. linearis*, but overlaps with *C. q. intermedia* in Lane County, Oregon (Gould, 1942). Within *C. quamash*, these three subspecies are the most difficult to separate using morphology. Leaves of *C. q. maxima* tend to be slightly glaucous, while leaves of the other two subspecies are not glaucous (Gould, 1942; Ranker & Hogan, 2002). Connivent tepal withering at senescence distinguishes *C. q. linearis* from all other taxa in the *C. q. azurea+* clade, whose tepals wither separately (Gould, 1942). Relatively pale flowers have been used to distinguish *C. q. intermedia* from *C. q. maxima* and *C. q. linearis*, but floral color is variable for *C. q. maxima* (noted above). Overall, the morphology of *C. q. intermedia* falls within the known ranges of *C. q. maxima*.

Earlier phylogenies had no well-supported resolution within this subclade (Archibald et al., 2015; Fishbein et al., 2010). Our analyses did not strongly infer relationships among most populations of *C. q. maxima*, but they did resolve a strongly supported *C. q. linearis* clade and a well-supported *C. q. intermedia* clade, both in a polytomy with *C. q. maxima*. The chloroplast tree of Fishbein et al. (2010) also supported the monophyly of *C. q. linearis*, but with just 0.67 posterior probability, while *C. q. intermedia* was weakly supported as not monophyletic.

Given its greater phylogenetic and morphological distinctiveness, and its geographic isolation, we support maintaining *C. q. linearis* as a subspecies, pending further study of this diverse subclade. Due to the noted lack of a clear morphological division between *C. q. intermedia* and *C. q. maxima*, and weaker phylogenetic evidence separating these subspecies, we propose this nomenclatural synonymy.

***Camassia quamash*** (Pursh) Greene **subsp.** ***maxima*** Gould. Amer. Midl. Nat. 1942. 28:732–733.

*Camassia quamash* (Pursh) Greene subsp. *intermedia* Gould. Amer. Midl. Nat. 1942. 28:734–735.

### The subspecies of *C. quamash* east of the Cascades (the *C. q. breviflora*+ clade) and a link to *C. cusickii*

As noted above, the second major clade of *C. quamash* included *C. q. breviflora*, *C. q. quamash*, and *C. q. utahensis* (Figs. 4 and 5). Farthest west from the other two and with the largest latitudinal range is *C. q. breviflora*, distributed from Washington to California. East of that range is *C. q. quamash*, distributed north of *C. q. utahensis* (Ranker & Hogan, 2002). Two of the sampled populations of *C. q. utahensis* fell within this *C. q. breviflora*+ clade (*C. q. utahensis*_CC, RCF), but two others were sister to *C. cusickii* (*C. q. utahensis*_CM, SR), separate from the rest of their species. Also occurring east of the Cascades, *C. cusickii* was once considered endemic to Oregon but additionally occurs across the border in Idaho (Adams County) and has disjunct populations in Washington (Kephart, Kephart & Robinson, 2019; Kephart, 2018).

Several morphological traits distinguish *C. quamash* and *C. cusickii*. Bulbs of *C. cusickii* are ellipsoid and clustered, while those of *C. quamash* are globose and rarely clustered. There are also rarely fewer than 10 leaves for *C. cusickii* and usually fewer than 10 for *C. quamash* (Kephart, 2018; Ranker & Hogan, 2002). Jewell (1978) noted differences in seed shape and indumentum, and that *C. cusickii* often has a densely flowered raceme, whereas *C. quamash* is more loosely flowered. A specific difference with *C. q. utahensis* may be tepal withering, which is often separate in *C. cusickii* and connivent in *C. q. utahensis* (Ranker & Hogan, 2002). Comparing morphology across the three subspecies of *C. quamash* in the *C. q. breviflora*+ clade, connivent tepals are also found in *C. q. breviflora*, but not in *C. q. quamash*, while a larger plant stature (20–70 cm) and distinctly bilateral flowers link *C. q. utahensis* and *C. q. quamash*, compared to the shorter *C. q. breviflora* (10–50 cm) with variable floral symmetry. For the latter two traits, *C. cusickii* has larger plants (50–90 cm) and variable floral symmetry (Kephart, 2015).

Jewell (1978) conducted a morphological, ecological, and flavonoid comparison of *C. cusickii* and *C. quamash*. He recommended that the two be maintained as separate species but noted intermediate specimens in the area of our *C. q. utahensis*_SR population, which fell in a clade sister to *C. cusickii* in our phylogenies. Jewell (1978) stated “Whereas these plants remain referrable to *C. quamash* var. *utahensis* in all the keys, they are intermediate in phenotypic expression between *C. cusickii* and *C. quamash* var. *utahensis*. This may presumably result from past hybridization and subsequent introgression”. The *C. q. utahensis*_CM population, which also clustered with *C. cusickii* in our and other analyses (Archibald et al., 2015; Fishbein et al., 2010), is only ∼10 km from SR.

Looking across our sampled populations, three of *C. q. utahensis* are within 15–23 km of our cluster of sampled populations of *C. cusickii* (Fig. 2), including the two that grouped with *C. cusickii* in the phylogeny (CM and SR), and one that did not (RCF; Figs. 4 and 5). The other population of *C. q. utahensis* (population CC) in the *C. quamash* clade is ∼520 km from this cluster of populations. Prior phylogenetic analyses differed in population sampling for *C. q. utahensis* and DNA regions used, with differences in some details of their inferences (Archibald et al., 2015; Fishbein et al., 2010). In all cases, some populations of *C. q. utahensis* group with *C. cusickii*, and others group with *C. quamash* near to *C. q. breviflora* and *C. q. quamash*. Fishbein et al. (2010) sampled three populations of *C. q. utahensis* that are not included in this study. Those populations were over 100–200 km from our *C. cusickii* sites, both east and west, and each grouped with *C. quamash*. In all, many populations of these two species were geographically close enough for potential gene flow, although a well-defined geographic pattern did not emerge for populations of *C. q. utahensis* that grouped with *C. quamash versus* those that grouped with *C. cusickii*.

Considering the monophyly of taxa across these clades, both prior phylogenetic studies sampled the same two populations of *C. cusickii*, which were not monophyletic in both cases (Archibald et al., 2015; Fishbein et al., 2010). We added two populations and multiple individuals from each population and resolved a well-supported *C. cusickii* clade (Figs. 4 and 5). When forced to be monophyletic by subspecies-grouped SVD analyses, *C. q. utahensis* was sister to *C. cusickii* (Fig. 5). In regard to the other two taxa in the *C. q. breviflora*+ clade, the monophyly of *C. q. quamash* was supported in these and prior analyses (Archibald et al., 2015; Fishbein et al., 2010), and each included the type locality (WP). The monophyly of *C. q. breviflora* was not fully supported due to a potential link between population CCT and the *C. q. quamash* clade, and due to outliers discussed above in the *C. leichtlinii*+ clade (Figs. 4 and 5).

Population genetic and morphological work are in progress to resolve this difficult complex (S Mortimer et al., unpublished data, 2025). Further study is needed, particularly with *C. q. utahensis* to verify if the phylogenetic division of its populations reflects gene flow across species and to address its lack of cohesion.

### A progenitor-derivative complex: the *C. scilloides*+ clade

The three potential taxa of *Camassia* that occur in the Midwest and farther south in the US (Fig. 2; Merritt, 2021; Ranker & Hogan, 2002) formed a strongly supported clade (*C. scilloides*+) that was sister to the *C. quamash*–*C. cusickii* clade (Figs. 4–6). This result was consistent across ML and SVD analyses, but it differs from previous inferences. The chloroplast trees of Fishbein et al. (2010) and Archibald et al. (2015) strongly inferred the *C. scilloides*+ clade as sister to a *C. q. breviflora*+ clade, nested within *C. quamash*. Separate ITS analyses of Archibald et al. (2015) instead placed *C. scilloides*+ as sister to all remaining *Camassia*, except *C. howellii*, but with variable support (Jackknife = 56, BI posterior probability = 0.92). Combined ITS and chloroplast analyses in Archibald et al. (2015) were consistent with the chloroplast resolution. Geographically, the *C. q. breviflora*+ clade does contain the easternmost subspecies of *C. quamash*, but it is still separated by over 1,300 km from the more eastern *C. scilloides* complex (Ranker & Hogan, 2002). The pattern of relationships supported by our study is surprising in that it differs from both previous hypotheses, but it concurs with the taxonomy in keeping a largely monophyletic *C. quamash*.

The *C. scilloides* complex was initially recognized as just one species. Gould (1942) stated in his treatment of the genus that *C. scilloides* and *C. angusta* were “certainly not specifically distinct”, but that they might be recognizable as subspecies. Two later studies of this pair instead argued for recognition of *C. angusta*, based on differences in morphology, flowering season, and allozyme markers (Ranker & Schnabel, 1986; Steyermark, 1961), and both species were recognized in the Flora of North America (Ranker & Hogan, 2002). Ranker & Schnabel (1986) studied 9–11 populations of each species and found that they had “taxon-specific patterns of morphological and isozymic variation”, but had diverged little. The species differed statistically for six of nine morphological traits (despite considerable overlap). Phenological isolation was strong in sympatry: flowering in *C. angusta* begins 2–3 weeks after ending in *C. scilloides*, and this difference was generally maintained in common garden experiments. The geographic range and allozyme markers of *C. angusta* were largely subsets of those for *C. scilloides*. Ranker & Schnabel (1986) thus proposed these two species as a recent progenitor-derivative species pair. Our phylogenetic results supported this hypothesis, nesting a *C. angusta*–*C.* “glade” clade within lineages of *C. scilloides*. Our results also suggested genetic isolation between *C. scilloides* and *C. angusta.* We sampled five individuals from two sites where those species are sympatric; in all cases individuals were placed with members of their own species in the phylogeny (Figs. 4 and 5; *C. scilloides* and *C. angusta* from site AND, and *C. angusta* from site GGR; *C. scilloides*_GGR was not available for this study).

Proposed recently is *C.* “glade” (Merritt, 2021), a potential taxon that shares some traits with *C. angusta* and others with *C. scilloides*, along with its own unique features. For example, capsules are ovoid-ellipsoid in *C.* “glade” and *C. angusta*; whereas *C. scilloides* has subglobose capsules. The former two species also flower later in the spring compared to *C. scilloides* and both have tepals that become strongly connivent over the ovary and then dehisce following anthesis (Merritt, 2021). Like *C. scilloides*, *C.* “glade” has fewer sterile bracts and flowers relative to *C. angusta*, and its racemes are more tapered at the top. Variation in perianth color may be taxon specific, but further work is needed. The major distinction for *C.* “glade” is that it is endemic to glade habitats composed of edaphic grasslands with thin soils amid exposed bedrock outcrops, in contrast to the prairie, wooded, or riparian habitats of *C. angusta* and *C. scilloides*. The *C.* “glade” sites are all in Arkansas and include flat, seasonally wet shale glades, and seasonally wet rocky riverscour glades composed of shale and sandstone in the Ouachita Mountains and in Saline County (T Witsell, pers. comm., 2012; Merritt, 2021). Most of our analyses resolved *C.* “glade” as monophyletic and sister to *C. angusta* (Figs. 4 and 5), although some SVD analyses showed potential intermixing of populations of *C. angusta* and *C.* “glade”. The connection between the two was well supported by these data, as was their separation from *C. scilloides*, but without strong evidence for or against recognizing *C.* “glade” as a formal taxon separate from *C. angusta*. An ecological niche modeling study (B Merritt et al., unpublished data, 2025) and a microsatellite study (T Culley et al., unpublished data, 2024) of *C. scilloides*, *C. angusta*, and *C*. “glade” may support further consideration of *C.* “glade” taxonomically. A valuable next step would be to pursue a “common garden” experiment with the three purported taxa, to see if any of the distinctive morphological features of *C.* “glade” are phenotypic responses to its unique environment.

## Conclusions

Overall, these RADseq analyses provided a robust framework for continued study of morphology, population genetics, ecology, and reproductive factors in *Camassia*. We found strong support of many relationships from the population to species levels, more so at deeper levels of the phylogeny. Comparing results with use of varying parameters for homology assessment and allowance of missing data allowed us to emphasize conclusions that are robust to changes in parameter decisions. The high level of missing data was overwhelmed in many cases by phylogenetic signal to allow inference of relationships. Basally, both *C. howellii* and *C. leichtlinii* separated early from the rest of *Camassia*. We inferred two possible relationships between the two species, with support for either a sister relationship or a grade, depending on the optimality criterion used. Within the widespread *C. leichtlinii*, relationships among many populations were strongly supported and patterns of diversification for some subclades suggested a geographic component in their diversification. The eight subspecies of *C. quamash* largely formed a monophyletic group, although a few outliers fell with *C. leichtlinii* or *C. cusickii*. In particular, *C. q. utahensis* was confirmed as being divided into two clades on the trees, one within *C. quamash* and the other sister to *C. cusickii*, which was itself supported for the first time as being monophyletic. Further investigation is needed to determine the status of *C. q. utahensis*. Relationships among other subspecies of *C. quamash* were not fully resolved but often showed some support for subspecies recognition. However, we recommend synonymizing *C. q. intermedia* with *C. q. maxima*. Although it may be monophyletic, *C. q. intermedia* was encompassed within *C. q. maxima* both morphologically and phylogenetically. The disjunct complex of species farther to the east in North America was strongly resolved as a monophyletic *C. scilloides*+ clade sister to the *C. quamash*–*C. cusickii* clade. *Camassia angusta* was supported as a derivative of progenitor *C. scilloides*, while a potential new taxon and edaphic specialist (*C.* “glade”) appeared either sister to or intermixed with *C. angusta*. Overall, the genus includes examples of clear-cut discrete species, a putative unnamed taxon, and potential admixture across current taxonomic boundaries, even involving currently allopatric populations.

##  Supplemental Information

10.7717/peerj.20438/supp-1 Supplemental Information 1 Scripts used to demultiplex FastQ files

## Appendix I

**Table A1:** Individuals sampled for phylogenetic analyses. Individual codes include site code and individual number (with collection year for *Camassia* and *Hastingsia*). Voucher information includes collector(s), collection number, herbarium, and herbarium accession number(s), if available. Inapplicable fields for a given individual are indicated by a “–”. All specimens were collected in the United States unless noted otherwise.

Taxon	Individual	Locality^a^	Voucher
*Camassia angusta* (Engelm. & A. Gray) Blank.	AND_2M, 8M (2012)	Kansas, Anderson Prairie, 38.18142, -95.26469	Archibald and Macon 2011-5 (KANU 393270)
*Camassia angusta* (Engelm. & A. Gray) Blank.	DTP_1, 2, 3 (2013)	Texas, David Talbot Prairie, –	Kephart, Cartieri, and Culley s.n. (CINC –)
*Camassia angusta* (Engelm. & A. Gray) Blank.	GGR_5ex (2012)	Texas, Gamble Goose Refuge, 33.70012, -95.6115	Holmes, Singhurst, and Mink 14513 (BAYLU 007444)
*Camassia angusta* (Engelm. & A. Gray) Blank.	OSA_18 (2012)	Kansas, Osage Haymeadow, 38.7822, -95.67857	Archibald, King, and Sharkan 2012-4 (KANU 393280)
*Camassia angusta* (Engelm. & A. Gray) Blank.	OTT_1M, 2M (2011)	Indiana, Otterbein Prairie, –	Homoya and Dana 900614 (BUT 155014)
*Camassia* “glade”	BIS_T11, T12 (2012)	Arkansas, Bismark, –,	Witsell, Kephart, Culley, and Cartieri 12-0199 (WILLU-01088)
*Camassia* “glade”	DLC_2 (2012)	Arkansas, Dry Lost Creek, –,	Witsell, Kephart, Culley, and Cartieri 12-0189 (WILLU-01086)
*Camassia cusickii* S. Watson	HC_7M, 8 (7M-2015, 8-2012)	Oregon, Hell’s Canyon, 45.126083, -116.836117	S. Kephart and J. Kephart 600 (WILLU-01063 )
*Camassia cusickii* S. Watson	IHC_6m, 13M, 19M (2015)	Idaho, Idaho Hell’s Canyon, 44.9905639, -116.8505833	Mortimer, Kephart, Parker, and Zimmer 2015-7 (OSC-V-269825, 269826)
*Camassia cusickii* S. Watson	P86_C7, 34 (2015)	Oregon, Pine Creek Hwy 86, 44.935567, -116.899736	Mortimer, Kephart, Parker, and Zimmer 2015-11 (OSC-V-270411, 270412)
*Camassia cusickii* S. Watson	PC_5, 16 (2006)	Oregon, Pine Creek, 45.0578, -116.9035	Kephart 805 (WILLU-01076)
*Camassia howellii* S. Watson	CCH_1, 6, 14, 15 (2013)	California, Cottage Creek Headquarters, –,	Janeway and Kephart 11138 (WILLU-01094)
*Camassia howellii* S. Watson	HRA_8, 12, 15 (2006)	Oregon, Hugo Rest Area, –,	Dennis and Kephart 101 (WILLU-01066)
*Camassia howellii* S. Watson	HUBS_16, 23 (2013)	Oregon, Hugo Bend Site, –,	Kephart, Sultany, and Dennis 588 (WILLU-01065)
*Camassia howellii* S. Watson	WHM_1M, 20M, 24M (2015)	Oregon, Weiss Hill Meadow, –,	Ahart, S. Kephart, and J. Kephart (WILLU-01096)
*Camassia leichtlinii* ssp. *leichtlinii* (Baker) S. Watson	GL_4M (2007)	Oregon, Glide, 43.301217, -123.225967	Kephart 578 (WILLU-01102)
*Camassia leichtlinii* ssp. *leichtlinii* (Baker) S. Watson	PS_19 (2007)	Oregon, Popcorn Swale, 43.301317, -123.225967	Kotaich and Kephart 105 (WILLU-01031)
*Camassia leichtlinii* ssp. *suksdorfii* (Greenman) Gould	BFV_L24, 5, 15 (2006)	California, Butterfly Valley, 40.012283, -120.994119	Kephart and Theiss 604 (WILLU-01058 )
*Camassia leichtlinii* ssp. *suksdorfii* (Greenman) Gould	BRM_1, 10 (2013)	Oregon, Boulder Ridge Meadow, 44.731716, -122.033447	Barosh and Kephart s.n. (WILLU-01079)
*Camassia leichtlinii* ssp. *suksdorfii* (Greenman) Gould	DDL_7 (2013)	Oregon, Detroit Dam Lower Rd, 44.729496, -122.261424	Kephart s.n. (OSC-V-270437)
*Camassia leichtlinii* ssp. *suksdorfii* (Greenman) Gould	DPK_12, 13, 14 (2004)	Canada, British Columbia, Mt. Douglas Park, 49.49265, -123.35035	Allen 1311 (WILLU-01036)
*Camassia leichtlinii* ssp. *suksdorfii* (Greenman) Gould	FC_Op21, WS12 (2008)	Oregon, Fruitland Creek, 44.914334, -122.938023	LaDouceur, Dick, and Kephart s.n. (WILLU-01009)
*Camassia leichtlinii* ssp. *suksdorfii* (Greenman) Gould	FG_L8 (2009)	Oregon, Fairgrounds Oak Grove, 44.960407, -123.0123953	S. Kephart, Donne, and J. Kephart s.n. (OSC-V-270432)
*Camassia leichtlinii* ssp. *suksdorfii* (Greenman) Gould	GCM_2M, 20M (2014)	California, Gurnsey Creek, 40.30741, -121.42474	Kephart and Pheil s.n. (WILLU-01062)
*Camassia leichtlinii* ssp. *suksdorfii* (Greenman) Gould	HEM_2, 3 (2013)	Oregon, Hemlock Lake, 43.1879166, -122.69655	Barosh, Cunningham, Glotherlery, Fogerty, and Kephart s.n. (WILLU-01077)
*Camassia leichtlinii* ssp. *suksdorfii* (Greenman) Gould	HUBS_1M (2012)	Oregon, Hugo Bend Site, 42.580563, -123.374306	Kephart, Dennis, and Sultany 589 (WILLU-01038)
*Camassia leichtlinii* ssp. *suksdorfii* (Greenman) Gould	KP_7M, 23M (2015)	Oregon, Kingston Prairie, 44.775903, -122.744819	Mortimer, Kephart, Parker, and Zimmer 2015-1 (WILLU-01109)
*Camassia leichtlinii* ssp. *suksdorfii* (Greenman) Gould	MDF_V, 3, 6 (2006)	Oregon, Mountain Dawn Farm, –	Kephart 583 (WILLU-01028)
*Camassia leichtlinii* ssp. *suksdorfii* (Greenman) Gould	PHR_1M, 14M, 22M (2015)	California, Philbrook Road, 40.03111799, -121.4891919	Parker, Kephart, and Mortimer 2015-12 (WILLU-01049)
*Camassia leichtlinii* ssp. *suksdorfii* (Greenman) Gould	TNC_8, L78 (8-2001, L78-2015)	Oregon, TNC-WLinn Preserve, 45.3612833, -122.61625	Kephart 580 (WILLU-01052)
*Camassia quamash* ssp. *azurea* (A.Heller) Gould	MM_2, 3 (2007)	Washington, Mima Mounds, 46.904633, -123.049567	Swift s.n. (WILLU-01022)
*Camassia quamash* ssp. *azurea* (A.Heller) Gould	WH_1, 2 (2008)	Washington, Wolf Haven, 46.90335, -122.848348	Kotaich 100 (WILLU-01034)
*Camassia quamash* ssp. *azurea* (A. Heller) Gould	SCR_15M, 19M (2015)	Washington, Scatter Creek Wildlife Refuge, 46.837971, -122.999757	Mortimer and Kephart 2015-4 (WILLU-01098)
*Camassia quamash* ssp. *breviflora* Gould	BOG_1M, 2M (2013)	California, Bogard Meadow, 40.540468, -121.108655	S. Kephart and J. Kephart 723 (WILLU-01043)
*Camassia quamash* ssp. *breviflora* Gould	CCT_4M, 9M, 23P (2013)	Washington, Catherine Creek Trail, 45.709816, -121.3619	S. Kephart and J. Kephart 675 (WILLU-01081)
*Camassia quamash* ssp. *breviflora* Gould	DL_4, 15 (2007)	California, Donner Lake, 39.3226, -120.24685	Dennis and Kephart s.n. (WILLU-01006)
*Camassia quamash* ssp. *breviflora* Gould	PPR_V, 7 (2008)	Oregon, Pigeon Prairie, 44.716735, -121.987843	Kotaich and Kephart s.n. (WILLU-01047)
*Camassia quamash* ssp. *breviflora* Gould	SHM_1, 3, 10, 15, 20 (2006)	California, Sagehen Meadow, 39.430217, -120.239651	Kephart 525 (WILLU-01025)
*Camassia quamash* ssp. *breviflora* Gould	ZUM_3, 8 (2008)	Oregon, Zumwalt Prairie, 45.55966, -116.986354	Kephart 612 (WILLU-01033)
*Camassia quamash* ssp. *intermedia* Gould	CG_11, 13, 14 (2008)	Oregon, Cottage Grove, 43.758, -123.095	Kotaich 104 (OSC-V-270317)
*Camassia quamash* ssp. *intermedia* Gould	PS_1, 1H (1-2007, 1H-2012)	Oregon, Popcorn Swale, 43.301317, -123.225967	Dennis 102 (WILLU-01030)
*Camassia quamash* ssp. *linearis* Gould	TB_1M, 3m, 9m (1M-2012, 3m,9m-2007)	California, Table Bluff, 40.6953, -124.2706	Mesler 758-A (240109)
*Camassia quamash* ssp. *maxima* Gould	BPP_A4, Q5, Q25 (2001)	Oregon, Bush Pasture Park, 44.929923, 123.035564	Torre 211 (WILLU-01054)
*Camassia quamash* ssp. *maxima* Gould	BVO_9 (2011)	Oregon, Bridal Veil Overlook, 45.554544, -122.183707	Kephart s.n. (WILLU-01075)
*Camassia quamash* ssp. *maxima* Gould	CGO_13, 17, 18 (2013)	Oregon, Columbia Gorge Overlook, 45.565669, -122.16353	J. Kephart and S. Kephart s.n. (OSC –)
*Camassia quamash* ssp. *maxima* Gould	FG_Q8 (2012)	Oregon, Fairgrounds Oak Grove, 44.960206, -123.013408	Mortimer, Busse, and Kephart s.n. (OSC-V-270433)
*Camassia quamash* ssp. *maxima* Gould	HEB_16 (2009)	Oregon, Mt. Hebo, 45.22385, -123.73625	Kephart 605 (WILLU-01039)
*Camassia quamash* ssp. *maxima* Gould	KP_1M, 12M, 22M (2015)	Oregon, Kingston Prairie, 44.777224, -122.744934	Mortimer, Kephart, Parker, and Zimmer 2015-2 (WILLU-01108)
*Camassia quamash* ssp. *maxima* Gould	LCP_C2, C8, F2 (2005)	Washington, LaCamas Prairie, 45.60158, -122.39921	Kephart and McClelland s.n. (WILLU-01061)
*Camassia quamash* ssp. *maxima* Gould	MDF_4M, 16M (2006?)	Oregon, Mountain Dawn Farm, 44.755566, -122.639753	Kephart s.n. (WILLU-01017)
*Camassia quamash* ssp. *maxima* Gould	SBH_18M (2014)	Washington, South Bald Hills Preserve, 46.81881, -122.4402	Kephart, McMillan, and Theiss (WILLU-01073)
*Camassia quamash* ssp. *maxima* Gould	SS_3, 4M, 6M (3-2006, 4M,6M-2014)	Oregon, 17th & Sunnyview, 44.95555, -123.00825	Uyeda 210 (WILLU-01003)
*Camassia quamash* ssp. *maxima* Gould	TNC_10, 12, 100 (10,12-2001, 100-2015)	Oregon, TNC-WLinn Camassia Preserve, 45.361283, -122.61625	Kephart 581 (WILLU-01053)
*Camassia quamash* ssp. *maxima* Gould	VIC_Q5, Q6, Q11 (2004)	Canada, British Columbia, University of Victoria, 48.460967, -123.3189	Allen 1310 (WILLU-01035)
*Camassia quamash* ssp. *quamash* (Pursh) Greene	MTK_1, 4, 7, 15 (2008)	Montana, Pattee Canyon, 46.860283, -113.88695	Snustad-Clark s.n. (WILLU-01060)
*Camassia quamash* ssp. *quamash* (Pursh) Greene	WP_9, 18, 22 (2008)	Idaho, Weippe Prairie, 46.349633, -115.9252	Kotaich and Kephart 111 (WILLU-01011)
*Camassia quamash* ssp. *utahensis* Gould	CC_8, 11 (2011)	Utah, Cherry Creek, 41.931861, -111.77944	Wolf 924 (WILLU-01029)
*Camassia quamash* ssp. *utahensis* Gould	CM_1, 3 (2008)	Oregon, Carson Meadow, 44.922265, -117.190783	Kotaich 123 (WILLU-01008)
*Camassia quamash* ssp. *utahensis* Gould	RCF_Q80, Q82 (2015)	Oregon, Rocky Comfort Flat, 44.961825, -116.6649417	Parker and Kephart s.n. (OSC-V-270413)
*Camassia quamash* ssp. *utahensis* Gould	SR_21 (2008)	Oregon, Sag Road N, 44.854977, -117.097838	Kotaich and Kephart 121 (WILLU-01020)
*Camassia quamash* ssp. *walpolei* (Piper) Gould	HM_1M, 2M, 11M (2013)	Oregon, Hogue Meadow, 42.0568, -123.62955	Archibald, Theiss, and Kephart 2013-s.n. (WILLU-50103)
*Camassia quamash* ssp. *walpolei* (Piper) Gould	HPR_3M, 18M (2014)	Oregon, Hyatt Prairie Road, 42.26299, -122.44927	J. Kephart and S. Kephart s.n. (WILLU-01105)
*Camassia quamash* ssp. *walpolei* (Piper) Gould	KR_1, 2, 15 (2007)	Oregon, Ken Rose, –	Dennis and Kephart 113 (WILLU-01042)
*Camassia scilloides* (Rafinesque) Cory	AND_12, 20 (2012)	Kansas, Anderson Prairie, 38.17262, -95.25349	Archibald and King 2012-2 (KANU 393278)
*Camassia scilloides* (Rafinesque) Cory	BAR_1, 12, 17 (2012)	Kansas, Barnhart Prairie, 39.23107, -95.02493	Archibald 2011-4 (KANU 393269)
*Camassia scilloides* (Rafinesque) Cory	BIE_5, 11 (2011)	Indiana, Biesecker Prairie, 41.4178, -87.468283	Schnabel s.n. (WILLU-01007)
*Camassia scilloides* (Rafinesque) Cory	CSP_1, 14 (2013)	North Carolina, Camassia Slopes Preserve, 36.324911, -77.459738	Peoples s.n. (OSC –)
*Camassia scilloides* (Rafinesque) Cory	HAL_11, 13 (2011)	Ohio, Hall’s Creek, 39.36731861, -84.15142363	Yadav and Culley 13-1001 (CINC –)
*Camassia scilloides* (Rafinesque) Cory	HOP_12 (2012)	Kansas, Hopewell Prairie, 38.45573, -94.92046	Archibald 2011-1 (KANU 393266)
*Camassia scilloides* (Rafinesque) Cory	PRO_14m, 19m (2012)	Indiana, Prophetstown, 40.51402, -86.80363	Cartieri, Harby, and Kephart (CINC –)
*Camassia scilloides* (Rafinesque) Cory	RFP_3, 4 (2012)	Indiana, Richardson Preserve, 39.30639, -84.60911	Cartieri (CINC –)
*Chlorogalum pomeridianum* var. *divaricatum* (Lindley) Hoover	AH2958_1, 3	California, UC Davis Bodega Marine Reserve, 38.3138055, -123.0681944	Howald and Sones 2958 (KANU 392053)
*Chlorogalum pomeridianum* var. *minus* Hoover	FCsn_a	California, Sunnyside, 39.89739, -122.97322	Callahan s.n. (–)
*Chlorogalum pomeridianum* var. *pomeridianum* (de Candolle) Kunth	MF5972_1	Oregon, 8 Dollar Mountain, 42.2315, -123.651	Fishbein 5972 (HPSU )
*Chlorogalum pomeridianum* var. *pomeridianum* (de Candolle) Kunth	JKN_2	California, Redding, 40.661488, -122.3084	Nelson 2013-009 (WILLU 50099)
*Hastingsia alba* (Durand) S. Watson	BFV_1M (2012)	California, Butterfly Valley, 40.01185, -120.99238	Kephart 661 (WILLU-01084)
*Hastingsia alba* (Durand) S. Watson	CLO_1 (2007)	California, Near Camp 18, east of Lake Oroville, Butte County, 39.624, -121.189117	Halpin 12 (–)
*Hastingsia alba* (Durand) S. Watson	DBT_1M, 17M, 24M (2012)	California, Darlingtonia Botanical Trail, 41.850778, -123.907972	Barosh and Theiss s.n. (WILLU-01100)
*Hastingsia alba* (Durand) S. Watson	H70_3M, 7M, 24M (2013)	California, Highway 70, 40.02452, -121.15084	Theiss s.n. (OSC –)
*Hastingsia alba* (Durand) S. Watson	RTR_3M, 20M, 21M (2013)	California, Rattlesnake Road, 40.46435, -123.18106	Ormond and Theiss s.n. (OSC –)
*Hastingsia alba* (Durand) S. Watson	WIL_1 (2007)	California, Willow lake, Lassen National Forest, 40.40685, -121.362717	Barosh, McGlothlen, Fogarty, and Cunningham (OSC-V-270395)
*Hastingsia atropurpurea* Becking	JMC_1M, 5M, 9M (2014)	Oregon, Jimmy Creek, 42.17416, -123.67464	McMillan, Theiss, and Kephart s.n. (OSC-V-270320)
*Hastingsia atropurpurea* Becking	WF_15 (2010)	Oregon, Woodcock Fen, 42.12755, -123.697	Kephart 640 (WILLU-01083)
*Hastingsia bracteosa* S. Watson	DAY_19M, 20M (2012)	Oregon, Day’s Gulch Fen, 42.223002, -123.70761	Barosh and Theiss s.n (OSC-V-270338)
*Hastingsia bracteosa* S. Watson	DCC_7BM, 12BM, 14M (2012)	Oregon, Deer Creek Center, 42.277183, -123.648136	Morse 1181b (–)
*Hastingsia bracteosa* S. Watson	GCR_1M, 3M, 23M (2012)	Oregon, Gold Canyon Road, 42.246783, -123.6378	Theiss, Pheil, McClelland, McMillan, and Kephart (OSC-V-270290)
*Hastingsia bracteosa* S. Watson	HF_1, 2 (2010)	Oregon, Howell’s Fen, 42.233263, -123.659023	Kephart 635 (WILLU-01104)
*Hastingsia serpentinicola* Becking^b^	DCC_4, 9 (2012)	Oregon, Deer Creek Center, 42.285217, -123.650533	Golub-Tse and Deatherage s.n. (OSC-V-027812)
*Hastingsia serpentinicola* Becking	GCR_1M, 10M (2012)	Oregon, Gold Canyon Road, 42.2463, -123.6372	Theiss, Pheil, McClelland, McMillan, and Kephart (OSC –)
*Hastingsia serpentinicola* Becking	HF_1, 2 (2010)	Oregon, Howell’s Fen, 42.233263, -123.659023	Kephart 642 (WILLU-01110)
*Hastingsia serpentinicola* Becking	RR_3, 7 (2010)	Oregon, Rough & Ready, 42.095183, -123.686733	Kephart 629 (WILLU-01082)
*Hastingsia serpentinicola* Becking	WF_17M (2012)	Oregon, Woodcock Fen, 42.12755, -123.697	Theiss s.n. (WILLU-05127)

**Notes.**

a Some coordinates have been withheld because the site is protected or on private land.

b *Hastingsia serpentinicola* is sometimes merged into *Hastingsia alba* (Durant) Watson and so these specimens may be annotated differently in some databases.

## Additional Information and Declarations

### Data Availability

The following information was supplied regarding data availability: The RADseq raw sequence data are available at NCBI BioProject: PRJNA847576
